# Supplementary material for: Structures reveal a key mechanism of WAVE regulatory complex activation by Rac1 GTPase
Source: Nat Commun. 2022 Sep 16;13:5444. doi: 10.1038/s41467-022-33174-3 (PMC9481577; doi:10.1038/s41467-022-33174-3)
Supplement: Supplementary file 1 — Supplementary Information [file 41467_2022_33174_MOESM1_ESM.pdf]

# -------- Supplementary Information --------

## *Structures reveal a key mechanism of WAVE Regulatory Complex activation by Rac1 GTPase*

**Supplementary Table 1. DNA constructs and WRC assemblies used in this study**

| Name | Description | Identifier | Source/reference |
|---|---|---|---|
| | **Individual proteins and WRC subunits** | | |
| Sra1 | His6-Tev-hSra1 (1-1253, full length) in pAV5a vector, His6-Tev finally removed | pYS1 | (Ismail et al., 2009)[1] |
| $Sra1^{D\text{-}Rac1}$ | His6-Tev-Sra1-$(GGS)_4$-$Rac1^{Q61L/P29S}$ (1-188) in pAV5a vector, His6-Tev finally removed | pYS11 | This study |
| $Sra1^{A\text{-}Rac1}$ | His6-Tev-Sra1 Y423-[$(GGS)_6$-$Rac1^{P29S}$ (1-188)-$(GS)_6$)]-S424 (Rac1 is inserted in a loop of Sra1 between Y423/S424), in pAV5a vector, His6-Tev finally removed | pYS88 | This study |
| $Sra1^{AD\text{-}Rac1}$ | His6-Tev-Sra1 Y423-[$(GGS)_6$-$Rac1^{Q61L/P29S}$ (1-188)-$(GS)_6$)]-S424 -$(GGS)_4$-$Rac1^{P29S}$ (1-188), in pAV5a vector, His6-Tev finally removed | pYS191 | This study |
| $Sra1^{N183R}$ | N183R in Sra1 | pYS208 | This study |
| $Sra1^{S186M}$ | S186M in Sra1 | pYS209 | This study |
| $Sra1^{K189M}$ | K189M in Sra1 | pYS210 | This study |
| $Sra1^{Y108A}$ | Y108A in Sra1 | pYS206 | This study |
| $Sra1^{Y108H}$ | Y108H in Sra1 | pYS192 | This study |
| $Sra1^{N176W}$ | N176W in Sra1 | pYS207 | This study |
| $Sra1^{R87C,\ D\text{-}Rac1}$ | R87C in $Sra1^{D\text{-}Rac1}$ | pYS213 | This study |
| Nap1 | His6-Tev-hNap1 (1-1128, full length), in pAV5a vector, His6-Tev finally removed | pYS2 | (Ismail et al., 2009)[1] |
| WAVE1 (1-230) | MBP-Tev-hWAVE1 (1-230) in pMalC2Tev vector, MBP-Tev finally removed | pYS8 | (Chen et al., 2017)[2] |
| WAVE1 (1-230)-WCA | MBP-Tev-hWAVE1 (1-230)-$(GGS)_6$-WCA(485-559) in pMalC2Tev vector, MBP-Tev finally removed | pYS9 | (Chen et al., 2017)[2] |
| WAVE1(1-230)-Rac1 | MBP-Tev-WAVE1 (1-230)-$(GGS)_6$-$Rac1^{Q61L/P29S}$ (1-188) in pMalC2Tev vector, MBP-Tev finally removed | cbyd-131103-2 (AE9-2) | (Chen et al., 2017)[2] |
| $WAVE1^{\Delta PPP}$ | $^{131}$PPPLNI$^{136}$ replaced by $(GS)_3$ in WAVE1 (1-230)-WCA | pYL17 | This study |
| $WAVE1^{Y151E}$ | Y151E in WAVE1 (1-230)-WCA | pYS221 | This study |
| Abi2 (1-158) | MBP-Tev-hAbi2 (1-158) in pMalC2Tev vector, MBP-Tev finally removed | pYS3 | (Ismail et al., 2009)[1] |
| HSPC300 | MBP-Tev-hHSPC300 (1-79, full length) in pMalC2Tev vector, MBP-Tev finally removed | pYS4 | (Ismail et al., 2009)[1] |
| WCA | hWAVE1(485-559) in pET11a vector | pYS194 | (Ismail et al., 2009)[1] |
| GST-$Rac1^{P29S}$ | GST-Tev-$Rac1^{P29S}$(1-188) in pGEXTev vector | pTB93 | (Chen et al., 2017)[2] |
| GST-$Rac1^{QP}$ | GST-Tev-$Rac1^{Q61L/P29S}$(1-188) in pGEXTev vector | pYS7 | (Chen et al., 2017)[2] |
| Untagged Rac1 | $Rac1^{Q61L/P29S}$(1-188) in pET11a vector | pYS69 | This study |
| EGFP-mCyfip1 | EGFP-mCyfip1 in pEGFP vector | pMS1 | (Schaks et al., 2018)[3] |
| EGFP-$mCyfip1^{C179R}$ | EGFP-$mCyfip1^{C179R}$ in pEGFP vector | pMS2 | (Schaks et al., 2018)[3] |
| EGFP-$mCyfip1^{Y108H}$ | EGFP-$mCyfip1^{Y108H}$ in pEGFP vector | pMS97 | (Schaks et al., 2020)[4] |
| EGFP-$mCyfip1^{Y108A}$ | EGFP-$mCyfip1^{Y108A}$ in pEGFP vector | pMS131 | This study |
| EGFP-$mCyfip1^{N176W}$ | EGFP-$mCyfip1^{N176W}$ in pEGFP vector | pMS132 | This study |

| | | | |
|---|---|---|---|
| EGFP-mCyfip1$^{N183R}$ | EGFP-mCyfip1$^{N183R}$ in pEGFP vector | pMS133 | This study |
| EGFP-mCyfip1$^{S186M}$ | EGFP-mCyfip1$^{S186M}$ in pEGFP vector | pMS134 | This study |
| EGFP-mCyfip1$^{K189M}$ | EGFP-mCyfip1$^{K189M}$ in pEGFP vector | pMS135 | This study |
| | **Assembled WRC (refer to the above table for subunit information)** | | |
| WRC$^{230WCA}$, or WRC$^{apo}$ | Sra1, Nap1, WAVE1 (1-230)-WCA, Abi2 (1-158), and HSPC300 | / | (Chen et al., 2017)[2] |
| WRC$^{D\text{-}Rac1}$ | Sra1$^{D\text{-}Rac1}$, Nap1, WAVE1 (1-230)-WCA, Abi2 (1-158), and HSPC300 | / | This study |
| WRC$^{A\text{-}Rac1}$ | Sra1$^{A\text{-}Rac1}$, Nap1, WAVE1 (1-230)-WCA, Abi2 (1-158), and HSPC300 | / | This study |
| WRC$^{AD\text{-}Rac1}$ | Sra1$^{AD\text{-}Rac1}$, Nap1, WAVE1 (1-230)-WCA, Abi2 (1-158), and HSPC300 | / | This study |
| WRC$^{N183R}$ | Sra1$^{N183R}$, Nap1, WAVE1 (1-230)-WCA, Abi2 (1-158), and HSPC300 | / | This study |
| WRC$^{S186M}$ | Sra1$^{S186M}$, Nap1, WAVE1 (1-230)-WCA, Abi2 (1-158), and HSPC300 | / | This study |
| WRC$^{K189M}$ | Sra1$^{K189M}$, Nap1, WAVE1 (1-230)-WCA, Abi2 (1-158), and HSPC300 | / | This study |
| WRC$^{Y108A}$ | Sra1$^{Y108A}$, Nap1, WAVE1 (1-230)-WCA, Abi2 (1-158), and HSPC300 | / | This study |
| WRC$^{Y108H}$ | Sra1$^{Y108H}$, Nap1, WAVE1 (1-230)-WCA, Abi2 (1-158), and HSPC300 | / | This study |
| WRC$^{N176W}$ | Sra1$^{N176W}$, Nap1, WAVE1 (1-230)-WCA, Abi2 (1-158), and HSPC300 | / | This study |
| WRC$^{D\text{-}Rac1,\ R87C}$ | Sra1$^{R87C,\ D\text{-}Rac1}$, Nap1, WAVE1 (1-230)-WCA, Abi2 (1-158), and HSPC300 | / | This study |
| WRC$^{D\text{-}Rac1,\ \Delta PPP}$ | Sra1$^{D\text{-}Rac1}$, Nap1, WAVE1$^{\Delta PPP}$, Abi2 (1-158), and HSPC300 | / | This study |
| WRC$^{D\text{-}Rac1,\ Y151E}$ | Sra1$^{D\text{-}Rac1}$, Nap1, WAVE1$^{Y151E}$, Abi2 (1-158), and HSPC300 | / | This study |
| WRC$^{230}$ | Sra1, Nap1, WAVE1 (1-230), Abi2 (1-158), and HSPC300 | / | (Chen et al., 2017)[2] |
| WRC$^{230,\ D\text{-}Rac1}$ | Sra1$^{D\text{-}Rac1}$, Nap1, WAVE1 (1-230), Abi2 (1-158), and HSPC300 | / | This study |
| WRC$^{230,\ A\text{-}Rac1}$ | Sra1$^{A\text{-}Rac1}$, Nap1, WAVE1 (1-230), Abi2 (1-158), and HSPC300 | / | This study |
| WRC$^{230\Delta WCA\text{-}Rac1}$ | Sra1, Nap1, WAVE1(1-230)-Rac1, Abi2 (1-158), and HSPC300 | / | (Chen et al., 2017)[2] |

**Supplementary Table 2. Amino acid sequences of recombinant proteins used in this study**
Only sequences in the final product (i.e., after protease cleavage to remove the affinity tag) are shown and are annotated by corresponding colors.

**>Sra1**
GAMAAQVTLEDALSNVDLLEELPLPDQQPCIEPPPSSLLYQPNFNTNFEDRNAFVTGIARYIEQATVHSSMNEMLEEGQEYAVMLYT
WRSCSRAIPQVKCNEQPNRVEIYEKTVEVLEPEVTKLMNFMYFQRNAIERFCGEVRRLCHAERRKDFVSEAYLITLGKFINMFAVLD
ELKNMKCSVKNDHSAYKRAAQFLRKMADPQSIQESQNLSMFLANHNKITQSLQQQLEVISGYEELLADIVNLCVDYYENRMYLTPSE
KHMLLKVMGFGLYLMDGSVSNIYKLDAKKRINLSKIDKYFKQLQVVPLFGDMQIELARYIKTSAHYEENKSRWTCTSSGSSPQYNIC
EQMIQIREDHMRFISELARYSNSEVVTGSGRQEAQKTDAEYRKLFDLALQGLQLLSQWSAHVMEVYSWKLVHPTDKYSNKDCPDSAE
EYERATRYNYTSEEKFALVEVIAMIKGLQVLMGRMESVFNHAIRHTVYAALQDFSQVTLREPLRQAIKKKKNVIQSVLQAIRKTVCD
WETGHEPFNDPALRGEKDPKSGFDIKVPRRAVGPSSTQLYMVRTMLESLIADKSGSKKTLRSSLEGPTILDIEKFHRESFFYTHLIN
FSETLQQCCDLSQLWFREFFLELTMGRRIQFPIEMSMPWILTDHILETKEASMMEYVLYSLDLYNDSAHYALTRFNKQFLYDEIEAE
VNLCFDQFVYKLADQIFAYYKVMAGSLLLDKRLRSECKNQGATIHLPPSNRYETLLKQRHVQLLGRSIDLNRLITQRVSAAMYKSLE
LAIGRFESEDLTSIVELDGLLEINRMTHKLLSRYLTLDGFDAMFREANHNVSAPYGRITLHVFWELNYDFLPNYCYNGSTNRFVRTV
LPFSQEFQRDKQPNAQPQYLHGSKALNLAYSSIYGSYRNFVGPPHFQVICRLLGYQGIAVVMEELLKVVKSLLQGTILQYVKTLMEV
MPKICRLPRHEYGSPGILEFFHHQLKDIVEYAELKTVCFQNLREVGNAILFCLLIEQSLSLEEVCDLLHAAPFQNILPRVHVKEGER
LDAKMKRLESKYAPLHLVPLIERLGTPQQIAIAREGDLLTKERLCCGLSMFEVILTRIRSFLDDPIWRGPLPSNGVMHVDECVEFHR
LWSAMQFVYCIPVGTHEFTVEQCFGDGLHWAGCMIIVLLGQQRRFAVLDFCYHLLKVQKHDGKDEIIKNVPLKKMVERIRKFQILND
EIITILDKYLKSGDGEGTPVEHVRCFQPPIHQSLASS

**>Sra1$^{D\text{-}Rac1}$**, or **Sra1-(GGS)$_4$-Rac1$^{Q61L/P29S}$(1-188)**
GAMAAQVTLEDALSNVDLLEELPLPDQQPCIEPPPSSLLYQPNFNTNFEDRNAFVTGIARYIEQATVHSSMNEMLEEGQEYAVMLYT
WRSCSRAIPQVKCNEQPNRVEIYEKTVEVLEPEVTKLMNFMYFQRNAIERFCGEVRRLCHAERRKDFVSEAYLITLGKFINMFAVLD
ELKNMKCSVKNDHSAYKRAAQFLRKMADPQSIQESQNLSMFLANHNKITQSLQQQLEVISGYEELLADIVNLCVDYYENRMYLTPSE
KHMLLKVMGFGLYLMDGSVSNIYKLDAKKRINLSKIDKYFKQLQVVPLFGDMQIELARYIKTSAHYEENKSRWTCTSSGSSPQYNIC
EQMIQIREDHMRFISELARYSNSEVVTGSGRQEAQKTDAEYRKLFDLALQGLQLLSQWSAHVMEVYSWKLVHPTDKYSNKDCPDSAE
EYERATRYNYTSEEKFALVEVIAMIKGLQVLMGRMESVFNHAIRHTVYAALQDFSQVTLREPLRQAIKKKKNVIQSVLQAIRKTVCD
WETGHEPFNDPALRGEKDPKSGFDIKVPRRAVGPSSTQLYMVRTMLESLIADKSGSKKTLRSSLEGPTILDIEKFHRESFFYTHLIN
FSETLQQCCDLSQLWFREFFLELTMGRRIQFPIEMSMPWILTDHILETKEASMMEYVLYSLDLYNDSAHYALTRFNKQFLYDEIEAE
VNLCFDQFVYKLADQIFAYYKVMAGSLLLDKRLRSECKNQGATIHLPPSNRYETLLKQRHVQLLGRSIDLNRLITQRVSAAMYKSLE
LAIGRFESEDLTSIVELDGLLEINRMTHKLLSRYLTLDGFDAMFREANHNVSAPYGRITLHVFWELNYDFLPNYCYNGSTNRFVRTV
LPFSQEFQRDKQPNAQPQYLHGSKALNLAYSSIYGSYRNFVGPPHFQVICRLLGYQGIAVVMEELLKVVKSLLQGTILQYVKTLMEV
MPKICRLPRHEYGSPGILEFFHHQLKDIVEYAELKTVCFQNLREVGNAILFCLLIEQSLSLEEVCDLLHAAPFQNILPRVHVKEGER
LDAKMKRLESKYAPLHLVPLIERLGTPQQIAIAREGDLLTKERLCCGLSMFEVILTRIRSFLDDPIWRGPLPSNGVMHVDECVEFHR
LWSAMQFVYCIPVGTHEFTVEQCFGDGLHWAGCMIIVLLGQQRRFAVLDFCYHLLKVQKHDGKDEIIKNVPLKKMVERIRKFQILND
EIITILDKYLKSGDGEGTPVEHVRCFQPPIHQSLASSGGSGGSGGSGGSMQAIKCVVVGDGAVGKTCLLISYTTNAFSGEYIPTVFD
NYSANVMVDGKPVNLGLWDTAGLEDYDRLRPLSYPQTDVFLICFSLVSPASFENVRAKWYPEVRHHCPNTPIILVGTKLDLRDDKDT
IEKLKEKKLTPITYPQGLAMAKEIGAVKYLECSALTQRGLKTVFDEAIRAVLCPPPVKKRKRK

**>Sra1$^{A\text{-}Rac1}$** or **Sra1 Y423-[(GGS)$_6$-Rac1$^{Q61L/P29S}$(1-188)-HRV3C-(GS)$_6$)]-S424**
GAMAAQVTLEDALSNVDLLEELPLPDQQPCIEPPPSSLLYQPNFNTNFEDRNAFVTGIARYIEQATVHSSMNEMLEEGQEYAVMLYT
WRSCSRAIPQVKCNEQPNRVEIYEKTVEVLEPEVTKLMNFMYFQRNAIERFCGEVRRLCHAERRKDFVSEAYLITLGKFINMFAVLD
ELKNMKCSVKNDHSAYKRAAQFLRKMADPQSIQESQNLSMFLANHNKITQSLQQQLEVISGYEELLADIVNLCVDYYENRMYLTPSE
KHMLLKVMGFGLYLMDGSVSNIYKLDAKKRINLSKIDKYFKQLQVVPLFGDMQIELARYIKTSAHYEENKSRWTCTSSGSSPQYNIC
EQMIQIREDHMRFISELARYSNSEVVTGSGRQEAQKTDAEYRKLFDLALQGLQLLSQWSAHVMEVYSWKLVHPTDKYGGSGGSGGSG
GSGGSGGSMQAIKCVVVGDGAVGKTCLLISYTTNAFSGEYIPTVFDNYSANVMVDGKPVNLGLWDTAGLEDYDRLRPLSYPQTDVFL
ICFSLVSPASFENVRAKWYPEVRHHCPNTPIILVGTKLDLRDDKDTIEKLKEKKLTPITYPQGLAMAKEIGAVKYLECSALTQRGLK
TVFDEAIRAVLCPPPVKKRKRKGSLEVLFQGPGSGSGSGSGSSNKDCPDSAEEYERATRYNYTSEEKFALVEVIAMIKGLQVLMGRM
ESVFNHAIRHTVYAALQDFSQVTLREPLRQAIKKKKNVIQSVLQAIRKTVCDWETGHEPFNDPALRGEKDPKSGFDIKVPRRAVGPS
STQLYMVRTMLESLIADKSGSKKTLRSSLEGPTILDIEKFHRESFFYTHLINFSETLQQCCDLSQLWFREFFLELTMGRRIQFPIEM
SMPWILTDHILETKEASMMEYVLYSLDLYNDSAHYALTRFNKQFLYDEIEAEVNLCFDQFVYKLADQIFAYYKVMAGSLLLDKRLRS
ECKNQGATIHLPPSNRYETLLKQRHVQLLGRSIDLNRLITQRVSAAMYKSLELAIGRFESEDLTSIVELDGLLEINRMTHKLLSRYL
TLDGFDAMFREANHNVSAPYGRITLHVFWELNYDFLPNYCYNGSTNRFVRTVLPFSQEFQRDKQPNAQPQYLHGSKALNLAYSSIYG
SYRNFVGPPHFQVICRLLGYQGIAVVMEELLKVVKSLLQGTILQYVKTLMEVMPKICRLPRHEYGSPGILEFFHHQLKDIVEYAELK
TVCFQNLREVGNAILFCLLIEQSLSLEEVCDLLHAAPFQNILPRVHVKEGERLDAKMKRLESKYAPLHLVPLIERLGTPQQIAIARE
GDLLTKERLCCGLSMFEVILTRIRSFLDDPIWRGPLPSNGVMHVDECVEFHRLWSAMQFVYCIPVGTHEFTVEQCFGDGLHWAGCMI
IVLLGQQRRFAVLDFCYHLLKVQKHDGKDEIIKNVPLKKMVERIRKFQILNDEIITILDKYLKSGDGEGTPVEHVRCFQPPIHQSLA
SS

**>Sra1$^{AD\text{-}Rac1}$ Sra1 Y423-[(GGS)$_6$-Rac1$^{Q61L/P29S}$(1-188)-(GS)$_6$)]-S424-(GGS)$_4$-Rac1$^{P29S}$(1-188)**. Note the DNA sequence for the second Rac1 (D site Rac1) is from a synthetic gene optimized for insect cell expression, which shares 76% sequence identity with the first Rac1 from the original human sequence to avoid unexpected recombination during cloning and protein expression.
GAMAAQVTLEDALSNVDLLEELPLPDQQPCIEPPPSSLLYQPNFNTNFEDRNAFVTGIARYIEQATVHSSMNEMLEEGQEYAVMLYT
WRSCSRAIPQVKCNEQPNRVEIYEKTVEVLEPEVTKLMNFMYFQRNAIERFCGEVRRLCHAERRKDFVSEAYLITLGKFINMFAVLD
ELKNMKCSVKNDHSAYKRAAQFLRKMADPQSIQESQNLSMFLANHNKITQSLQQQLEVISGYEELLADIVNLCVDYYENRMYLTPSE

KHMLLKVMGFGLYLMDGSVSNIYKLDAKKRINLSKIDKYFKQLQVVPLFGDMQIELARYIKTSAHYEENKSRWTCTSSGSSPQYNIC
EQMIQIREDHMRFISELARYSNSEVVTGSGRQEAQKTDAEYRKLFDLALQGLQLLSQWSAHVMEVYSWKLVHPTDKYGGSGGSGGSG
GSGGSGGSMQAIKCVVVGDGAVGKTCLLISYTTNAFSGEYIPTVFDNYSANVMVDGKPVNLGLWDTAGLEDYDRLRPLSYPQTDVFL
ICFSLVSPASFENVRAKWYPEVRHHCPNTPIILVGTKLDLRDDKDTIEKLKEKKLTPITYPQGLAMAKEIGAVKYLECSALTQRGLK
TVFDEAIRAVLCPPPVKKRKRKGSGSGSGSGSGSSNKDCPDSAEEYERATRYNYTSEEKFALVEVIAMIKGLQVLMGRMESVFNHAI
RHTVYAALQDFSQVTLREPLRQAIKKKKNVIQSVLQAIRKTVCDWETGHEPFNDPALRGEKDPKSGFDIKVPRRAVGPSSTQLYMVR
TMLESLIADKSGSKKTLRSSLEGPTILDIEKFHRESFFYTHLINFSETLQQCCDLSQLWFREFFLELTMGRRIQFPIEMSMPWILTD
HILETKEASMMEYVLYSLDLYNDSAHYALTRFNKQFLYDEIEAEVNLCFDQFVYKLADQIFAYYKVMAGSLLLDKRLRSECKNQGAT
IHLPPSNRYETLLKQRHVQLLGRSIDLNRLITQRVSAAMYKSLELAIGRFESEDLTSIVELDGLLEINRMTHKLLSRYLTLDGFDAM
FREANHNVSAPYGRITLHVFWELNYDFLPNYCYNGSTNRFVRTVLPFSQEFQRDKQPNAQPQYLHGSKALNLAYSSIYGSYRNFVGP
PHFQVICRLLGYQGIAVVMEELLKVVKSLLQGTILQYVKTLMEVMPKICRLPRHEYGSPGILEFFHHQLKDIVEYAELKTVCFQNLR
EVGNAILFCLLIEQSLSLEEVCDLLHAAPFQNILPRVHVKEGERLDAKMKRLESKYAPLHLVPLIERLGTPQQIAIAREGDLLTKER
LCCGLSMFEVILTRIRSFLDDPIWRGPLPSNGVMHVDECVEFHRLWSAMQFVYCIPVGTHEFTVEQCFGDGLHWAGCMIIVLLGQQR
RFAVLDFCYHLLKVQKHDGKDEIIKNVPLKKMVERIRKFQILNDEIITILDKYLKSGDGEGTPVEHVRCFQPPIHQSLASSGGSGGS
GGSGGSMQAIKCVVVGDGAVGKTCLLISYTTNAFSGEYIPTVFDNYSANVMVDGKPVNLGLWDTAGQEDYDRLRPLSYPQTDVFLIC
FSLVSPASFENVRAKWYPEVRHHCPNTPIILVGTKLDLRDDKDTIEKLKEKKLTPITYPQGLAMAKEIGAVKYLECSALTQRGLKTV
FDEAIRAVLCPPPVKKRKRK

**>Sra1$^{N183R}$**

GAMAAQVTLEDALSNVDLLEELPLPDQQPCIEPPPSSLLYQPNFNTNFEDRNAFVTGIARYIEQATVHSSMNEMLEEGQEYAVMLYT
WRSCSRAIPQVKCNEQPNRVEIYEKTVEVLEPEVTKLMNFMYFQRNAIERFCGEVRRLCHAERRKDFVSEAYLITLGKFINMFAVLD
ELKNMKCSVKRDHSAYKRAAQFLRKMADPQSIQESQNLSMFLANHNKITQSLQQQLEVISGYEELLADIVNLCVDYYENRMYLTPSE
KHMLLKVMGFGLYLMDGSVSNIYKLDAKKRINLSKIDKYFKQLQVVPLFGDMQIELARYIKTSAHYEENKSRWTCTSSGSSPQYNIC
EQMIQIREDHMRFISELARYSNSEVVTGSGRQEAQKTDAEYRKLFDLALQGLQLLSQWSAHVMEVYSWKLVHPTDKYSNKDCPDSAE
EYERATRYNYTSEEKFALVEVIAMIKGLQVLMGRMESVFNHAIRHTVYAALQDFSQVTLREPLRQAIKKKKNVIQSVLQAIRKTVCD
WETGHEPFNDPALRGEKDPKSGFDIKVPRRAVGPSSTQLYMVRTMLESLIADKSGSKKTLRSSLEGPTILDIEKFHRESFFYTHLIN
FSETLQQCCDLSQLWFREFFLELTMGRRIQFPIEMSMPWILTDHILETKEASMMEYVLYSLDLYNDSAHYALTRFNKQFLYDEIEAE
VNLCFDQFVYKLADQIFAYYKVMAGSLLLDKRLRSECKNQGATIHLPPSNRYETLLKQRHVQLLGRSIDLNRLITQRVSAAMYKSLE
LAIGRFESEDLTSIVELDGLLEINRMTHKLLSRYLTLDGFDAMFREANHNVSAPYGRITLHVFWELNYDFLPNYCYNGSTNRFVRTV
LPFSQEFQRDKQPNAQPQYLHGSKALNLAYSSIYGSYRNFVGPPHFQVICRLLGYQGIAVVMEELLKVVKSLLQGTILQYVKTLMEV
MPKICRLPRHEYGSPGILEFFHHQLKDIVEYAELKTVCFQNLREVGNAILFCLLIEQSLSLEEVCDLLHAAPFQNILPRVHVKEGER
LDAKMKRLESKYAPLHLVPLIERLGTPQQIAIAREGDLLTKERLCCGLSMFEVILTRIRSFLDDPIWRGPLPSNGVMHVDECVEFHR
LWSAMQFVYCIPVGTHEFTVEQCFGDGLHWAGCMIIVLLGQQRRFAVLDFCYHLLKVQKHDGKDEIIKNVPLKKMVERIRKFQILND
EIITILDKYLKSGDGEGTPVEHVRCFQPPIHQSLASS

**>Sra1$^{S186M}$**

GAMAAQVTLEDALSNVDLLEELPLPDQQPCIEPPPSSLLYQPNFNTNFEDRNAFVTGIARYIEQATVHSSMNEMLEEGQEYAVMLYT
WRSCSRAIPQVKCNEQPNRVEIYEKTVEVLEPEVTKLMNFMYFQRNAIERFCGEVRRLCHAERRKDFVSEAYLITLGKFINMFAVLD
ELKNMKCSVKNDHMAYKRAAQFLRKMADPQSIQESQNLSMFLANHNKITQSLQQQLEVISGYEELLADIVNLCVDYYENRMYLTPSE
KHMLLKVMGFGLYLMDGSVSNIYKLDAKKRINLSKIDKYFKQLQVVPLFGDMQIELARYIKTSAHYEENKSRWTCTSSGSSPQYNIC
EQMIQIREDHMRFISELARYSNSEVVTGSGRQEAQKTDAEYRKLFDLALQGLQLLSQWSAHVMEVYSWKLVHPTDKYSNKDCPDSAE
EYERATRYNYTSEEKFALVEVIAMIKGLQVLMGRMESVFNHAIRHTVYAALQDFSQVTLREPLRQAIKKKKNVIQSVLQAIRKTVCD
WETGHEPFNDPALRGEKDPKSGFDIKVPRRAVGPSSTQLYMVRTMLESLIADKSGSKKTLRSSLEGPTILDIEKFHRESFFYTHLIN
FSETLQQCCDLSQLWFREFFLELTMGRRIQFPIEMSMPWILTDHILETKEASMMEYVLYSLDLYNDSAHYALTRFNKQFLYDEIEAE
VNLCFDQFVYKLADQIFAYYKVMAGSLLLDKRLRSECKNQGATIHLPPSNRYETLLKQRHVQLLGRSIDLNRLITQRVSAAMYKSLE
LAIGRFESEDLTSIVELDGLLEINRMTHKLLSRYLTLDGFDAMFREANHNVSAPYGRITLHVFWELNYDFLPNYCYNGSTNRFVRTV
LPFSQEFQRDKQPNAQPQYLHGSKALNLAYSSIYGSYRNFVGPPHFQVICRLLGYQGIAVVMEELLKVVKSLLQGTILQYVKTLMEV
MPKICRLPRHEYGSPGILEFFHHQLKDIVEYAELKTVCFQNLREVGNAILFCLLIEQSLSLEEVCDLLHAAPFQNILPRVHVKEGER
LDAKMKRLESKYAPLHLVPLIERLGTPQQIAIAREGDLLTKERLCCGLSMFEVILTRIRSFLDDPIWRGPLPSNGVMHVDECVEFHR
LWSAMQFVYCIPVGTHEFTVEQCFGDGLHWAGCMIIVLLGQQRRFAVLDFCYHLLKVQKHDGKDEIIKNVPLKKMVERIRKFQILND
EIITILDKYLKSGDGEGTPVEHVRCFQPPIHQSLASS

**>Sra1$^{K189M}$**

GAMAAQVTLEDALSNVDLLEELPLPDQQPCIEPPPSSLLYQPNFNTNFEDRNAFVTGIARYIEQATVHSSMNEMLEEGQEYAVMLYT
WRSCSRAIPQVKCNEQPNRVEIYEKTVEVLEPEVTKLMNFMYFQRNAIERFCGEVRRLCHAERRKDFVSEAYLITLGKFINMFAVLD
ELKNMKCSVKNDHSAYMRAAQFLRKMADPQSIQESQNLSMFLANHNKITQSLQQQLEVISGYEELLADIVNLCVDYYENRMYLTPSE
KHMLLKVMGFGLYLMDGSVSNIYKLDAKKRINLSKIDKYFKQLQVVPLFGDMQIELARYIKTSAHYEENKSRWTCTSSGSSPQYNIC
EQMIQIREDHMRFISELARYSNSEVVTGSGRQEAQKTDAEYRKLFDLALQGLQLLSQWSAHVMEVYSWKLVHPTDKYSNKDCPDSAE
EYERATRYNYTSEEKFALVEVIAMIKGLQVLMGRMESVFNHAIRHTVYAALQDFSQVTLREPLRQAIKKKKNVIQSVLQAIRKTVCD
WETGHEPFNDPALRGEKDPKSGFDIKVPRRAVGPSSTQLYMVRTMLESLIADKSGSKKTLRSSLEGPTILDIEKFHRESFFYTHLIN
FSETLQQCCDLSQLWFREFFLELTMGRRIQFPIEMSMPWILTDHILETKEASMMEYVLYSLDLYNDSAHYALTRFNKQFLYDEIEAE
VNLCFDQFVYKLADQIFAYYKVMAGSLLLDKRLRSECKNQGATIHLPPSNRYETLLKQRHVQLLGRSIDLNRLITQRVSAAMYKSLE
LAIGRFESEDLTSIVELDGLLEINRMTHKLLSRYLTLDGFDAMFREANHNVSAPYGRITLHVFWELNYDFLPNYCYNGSTNRFVRTV
LPFSQEFQRDKQPNAQPQYLHGSKALNLAYSSIYGSYRNFVGPPHFQVICRLLGYQGIAVVMEELLKVVKSLLQGTILQYVKTLMEV
MPKICRLPRHEYGSPGILEFFHHQLKDIVEYAELKTVCFQNLREVGNAILFCLLIEQSLSLEEVCDLLHAAPFQNILPRVHVKEGER
LDAKMKRLESKYAPLHLVPLIERLGTPQQIAIAREGDLLTKERLCCGLSMFEVILTRIRSFLDDPIWRGPLPSNGVMHVDECVEFHR
LWSAMQFVYCIPVGTHEFTVEQCFGDGLHWAGCMIIVLLGQQRRFAVLDFCYHLLKVQKHDGKDEIIKNVPLKKMVERIRKFQILND
EIITILDKYLKSGDGEGTPVEHVRCFQPPIHQSLASS

**>Sra1$^{Y108A}$**

GAMAAQVTLEDALSNVDLLEELPLPDQQPCIEPPPSSLLYQPNFNTNFEDRNAFVTGIARYIEQATVHSSMNEMLEEGQEYAVMLYT
WRSCSRAIPQVKCNEQPNRVEIAEKTVEVLEPEVTKLMNFMYFQRNAIERFCGEVRRLCHAERRKDFVSEAYLITLGKFINMFAVLD

ELKNMKCSVKNDHSAYKRAAQFLRKMADPQSIQESQNLSMFLANHNKITQSLQQQLEVISGYEELLADIVNLCVDYYENRMYLTPSE
KHMLLKVMGFGLYLMDGSVSNIYKLDAKKRINLSKIDKYFKQLQVVPLFGDMQIELARYIKTSAHYEENKSRWTCTSSGSSPQYNIC
EQMIQIREDHMRFISELARYSNSEVVTGSGRQEAQKTDAEYRKLFDLALQGLQLLSQWSAHVMEVYSWKLVHPTDKYSNKDCPDSAE
EYERATRYNYTSEEKFALVEVIAMIKGLQVLMGRMESVFNHAIRHTVYAALQDFSQVTLREPLRQAIKKKKNVIQSVLQAIRKTVCD
WETGHEPFNDPALRGEKDPKSGFDIKVPRRAVGPSSTQLYMVRTMLESLIADKSGSKKTLRSSLEGPTILDIEKFHRESFFYTHLIN
FSETLQQCCDLSQLWFREFFLELTMGRRIQFPIEMSMPWILTDHILETKEASMMEYVLYSLDLYNDSAHYALTRFNKQFLYDEIEAE
VNLCFDQFVYKLADQIFAYYKVMAGSLLLDKRLRSECKNQGATIHLPPSNRYETLLKQRHVQLLGRSIDLNRLITQRVSAAMYKSLE
LAIGRFESEDLTSIVELDGLLEINRMTHKLLSRYLTLDGFDAMFREANHNVSAPYGRITLHVFWELNYDFLPNYCYNGSTNRFVRTV
LPFSQEFQRDKQPNAQPQYLHGSKALNLAYSSIYGSYRNFVGPPHFQVICRLLGYQGIAVVMEELLKVVKSLLQGTILQYVKTLMEV
MPKICRLPRHEYGSPGILEFFHHQLKDIVEYAELKTVCFQNLREVGNAILFCLLIEQSLSLEEVCDLLHAAPFQNILPRVHVKEGER
LDAKMKRLESKYAPLHLVPLIERLGTPQQIAIAREGDLLTKERLCCGLSMFEVILTRIRSFLDDPIWRGPLPSNGVMHVDECVEFHR
LWSAMQFVYCIPVGTHEFTVEQCFGDGLHWAGCMIIVLLGQQRRFAVLDFCYHLLKVQKHDGKDEIIKNVPLKKMVERIRKFQILND
EIITILDKYLKSGDGEGTPVEHVRCFQPPIHQSLASS

**>Sra1$^{Y108H}$**

GAMAAQVTLEDALSNVDLLEELPLPDQQPCIEPPPSSLLYQPNFNTNFEDRNAFVTGIARYIEQATVHSSMNEMLEEGQEYAVMLYT
WRSCSRAIPQVKCNEQPNRVEI**H**EKTVEVLEPEVTKLMNFMYFQRNAIERFCGEVRRLCHAERRKDFVSEAYLITLGKFINMFAVLD
ELKNMKCSVKNDHSAYKRAAQFLRKMADPQSIQESQNLSMFLANHNKITQSLQQQLEVISGYEELLADIVNLCVDYYENRMYLTPSE
KHMLLKVMGFGLYLMDGSVSNIYKLDAKKRINLSKIDKYFKQLQVVPLFGDMQIELARYIKTSAHYEENKSRWTCTSSGSSPQYNIC
EQMIQIREDHMRFISELARYSNSEVVTGSGRQEAQKTDAEYRKLFDLALQGLQLLSQWSAHVMEVYSWKLVHPTDKYSNKDCPDSAE
EYERATRYNYTSEEKFALVEVIAMIKGLQVLMGRMESVFNHAIRHTVYAALQDFSQVTLREPLRQAIKKKKNVIQSVLQAIRKTVCD
WETGHEPFNDPALRGEKDPKSGFDIKVPRRAVGPSSTQLYMVRTMLESLIADKSGSKKTLRSSLEGPTILDIEKFHRESFFYTHLIN
FSETLQQCCDLSQLWFREFFLELTMGRRIQFPIEMSMPWILTDHILETKEASMMEYVLYSLDLYNDSAHYALTRFNKQFLYDEIEAE
VNLCFDQFVYKLADQIFAYYKVMAGSLLLDKRLRSECKNQGATIHLPPSNRYETLLKQRHVQLLGRSIDLNRLITQRVSAAMYKSLE
LAIGRFESEDLTSIVELDGLLEINRMTHKLLSRYLTLDGFDAMFREANHNVSAPYGRITLHVFWELNYDFLPNYCYNGSTNRFVRTV
LPFSQEFQRDKQPNAQPQYLHGSKALNLAYSSIYGSYRNFVGPPHFQVICRLLGYQGIAVVMEELLKVVKSLLQGTILQYVKTLMEV
MPKICRLPRHEYGSPGILEFFHHQLKDIVEYAELKTVCFQNLREVGNAILFCLLIEQSLSLEEVCDLLHAAPFQNILPRVHVKEGER
LDAKMKRLESKYAPLHLVPLIERLGTPQQIAIAREGDLLTKERLCCGLSMFEVILTRIRSFLDDPIWRGPLPSNGVMHVDECVEFHR
LWSAMQFVYCIPVGTHEFTVEQCFGDGLHWAGCMIIVLLGQQRRFAVLDFCYHLLKVQKHDGKDEIIKNVPLKKMVERIRKFQILND
EIITILDKYLKSGDGEGTPVEHVRCFQPPIHQSLASS

**>Sra1$^{N176W}$**

GAMAAQVTLEDALSNVDLLEELPLPDQQPCIEPPPSSLLYQPNFNTNFEDRNAFVTGIARYIEQATVHSSMNEMLEEGQEYAVMLYT
WRSCSRAIPQVKCNEQPNRVEIYEKTVEVLEPEVTKLMNFMYFQRNAIERFCGEVRRLCHAERRKDFVSEAYLITLGKFINMFAVLD
ELK**W**MKCSVKNDHSAYKRAAQFLRKMADPQSIQESQNLSMFLANHNKITQSLQQQLEVISGYEELLADIVNLCVDYYENRMYLTPSE
KHMLLKVMGFGLYLMDGSVSNIYKLDAKKRINLSKIDKYFKQLQVVPLFGDMQIELARYIKTSAHYEENKSRWTCTSSGSSPQYNIC
EQMIQIREDHMRFISELARYSNSEVVTGSGRQEAQKTDAEYRKLFDLALQGLQLLSQWSAHVMEVYSWKLVHPTDKYSNKDCPDSAE
EYERATRYNYTSEEKFALVEVIAMIKGLQVLMGRMESVFNHAIRHTVYAALQDFSQVTLREPLRQAIKKKKNVIQSVLQAIRKTVCD
WETGHEPFNDPALRGEKDPKSGFDIKVPRRAVGPSSTQLYMVRTMLESLIADKSGSKKTLRSSLEGPTILDIEKFHRESFFYTHLIN
FSETLQQCCDLSQLWFREFFLELTMGRRIQFPIEMSMPWILTDHILETKEASMMEYVLYSLDLYNDSAHYALTRFNKQFLYDEIEAE
VNLCFDQFVYKLADQIFAYYKVMAGSLLLDKRLRSECKNQGATIHLPPSNRYETLLKQRHVQLLGRSIDLNRLITQRVSAAMYKSLE
LAIGRFESEDLTSIVELDGLLEINRMTHKLLSRYLTLDGFDAMFREANHNVSAPYGRITLHVFWELNYDFLPNYCYNGSTNRFVRTV
LPFSQEFQRDKQPNAQPQYLHGSKALNLAYSSIYGSYRNFVGPPHFQVICRLLGYQGIAVVMEELLKVVKSLLQGTILQYVKTLMEV
MPKICRLPRHEYGSPGILEFFHHQLKDIVEYAELKTVCFQNLREVGNAILFCLLIEQSLSLEEVCDLLHAAPFQNILPRVHVKEGER
LDAKMKRLESKYAPLHLVPLIERLGTPQQIAIAREGDLLTKERLCCGLSMFEVILTRIRSFLDDPIWRGPLPSNGVMHVDECVEFHR
LWSAMQFVYCIPVGTHEFTVEQCFGDGLHWAGCMIIVLLGQQRRFAVLDFCYHLLKVQKHDGKDEIIKNVPLKKMVERIRKFQILND
EIITILDKYLKSGDGEGTPVEHVRCFQPPIHQSLASS

**>Sra1$^{R87C, D\text{-}Rac1}$, or Sra1$^{R87C}$-(GGS)$_4$-Rac1$^{Q61L/P29S}$(1-188)**

GAMAAQVTLEDALSNVDLLEELPLPDQQPCIEPPPSSLLYQPNFNTNFEDRNAFVTGIARYIEQATVHSSMNEMLEEGQEYAVMLYT
W**C**SCSRAIPQVKCNEQPNRVEIYEKTVEVLEPEVTKLMNFMYFQRNAIERFCGEVRRLCHAERRKDFVSEAYLITLGKFINMFAVLD
ELKNMKCSVKNDHSAYKRAAQFLRKMADPQSIQESQNLSMFLANHNKITQSLQQQLEVISGYEELLADIVNLCVDYYENRMYLTPSE
KHMLLKVMGFGLYLMDGSVSNIYKLDAKKRINLSKIDKYFKQLQVVPLFGDMQIELARYIKTSAHYEENKSRWTCTSSGSSPQYNIC
EQMIQIREDHMRFISELARYSNSEVVTGSGRQEAQKTDAEYRKLFDLALQGLQLLSQWSAHVMEVYSWKLVHPTDKYSNKDCPDSAE
EYERATRYNYTSEEKFALVEVIAMIKGLQVLMGRMESVFNHAIRHTVYAALQDFSQVTLREPLRQAIKKKKNVIQSVLQAIRKTVCD
WETGHEPFNDPALRGEKDPKSGFDIKVPRRAVGPSSTQLYMVRTMLESLIADKSGSKKTLRSSLEGPTILDIEKFHRESFFYTHLIN
FSETLQQCCDLSQLWFREFFLELTMGRRIQFPIEMSMPWILTDHILETKEASMMEYVLYSLDLYNDSAHYALTRFNKQFLYDEIEAE
VNLCFDQFVYKLADQIFAYYKVMAGSLLLDKRLRSECKNQGATIHLPPSNRYETLLKQRHVQLLGRSIDLNRLITQRVSAAMYKSLE
LAIGRFESEDLTSIVELDGLLEINRMTHKLLSRYLTLDGFDAMFREANHNVSAPYGRITLHVFWELNYDFLPNYCYNGSTNRFVRTV
LPFSQEFQRDKQPNAQPQYLHGSKALNLAYSSIYGSYRNFVGPPHFQVICRLLGYQGIAVVMEELLKVVKSLLQGTILQYVKTLMEV
MPKICRLPRHEYGSPGILEFFHHQLKDIVEYAELKTVCFQNLREVGNAILFCLLIEQSLSLEEVCDLLHAAPFQNILPRVHVKEGER
LDAKMKRLESKYAPLHLVPLIERLGTPQQIAIAREGDLLTKERLCCGLSMFEVILTRIRSFLDDPIWRGPLPSNGVMHVDECVEFHR
LWSAMQFVYCIPVGTHEFTVEQCFGDGLHWAGCMIIVLLGQQRRFAVLDFCYHLLKVQKHDGKDEIIKNVPLKKMVERIRKFQILND
EIITILDKYLKSGDGEGTPVEHVRCFQPPIHQSLASS**GGSGGSGGSGGS**MQAIKCVVVGDGAVGKTCLLISYTTNAF**S**GEYIPTVFD
NYSANVMVDGKPVNLGLWDTAG**L**EDYDRLRPLSYPQTDVFLICFSLVSPASFENVRAKWYPEVRHHCPNTPIILVGTKLDLRDDKDT
IEKLKEKKLTPITYPQGLAMAKEIGAVKYLECSALTQRGLKTVFDEAIRAVLCPPPVKKRKRK

**>Nap1**

GAMSRSVLQPSQQKLAEKLTILNDRGVGMLTRLYNIKKACGDPKAKPSYLIDKNLESAVKFIVRKFPAVETRNNNQQLAQLQKEKSE
ILKNLALYYFTFVDVMEFKDHVCELLNTIDVCQVFFDITVNFDLTKNYLDLIITYTTLMILLSRIEERKAIIGLYNYAHEMTHGASD
REYPRLGQMIVDYENPLKKMMEEFVPHSKSLSDALISLQMVYPRRNLSADQWRNAQLLSLISAPSTMLNPAQSDTMPCEYLSLDAME

KWIIFGFILCHGILNTDATALNLWKLALQSSSCLSLFRDEVFHIHKAAEDLFVNIRGYNKRINDIRECKEAAVSHAGSMHRERRKFL
RSALKELATVLSDQPGLLGPKALFVFMALSFARDEIIWLLRHADNMPKKSADDFIDKHIAELIFYMEELRAHVRKYGPVMQRYYVQY
LSGFDAVVLNELVQNLSVCPEDESIIMSSFVNTMTSLSVKQVEDGEVFDFRGMRLDWFRLQAYTSVSKASLGLADHRELGKMMNTII
FHTKMVDSLVEMLVETSDLSIFCFYSRAFEKMFQQCLELPSQSRYSIAFPLLCTHFMSCTHELCPEERHHIGDRSLSLCNMFLDEMA
KQARNLITDICTEQCTLSDQLLPKHCAKTISQAVNKKSKKQTGKKGEPEREKPGVESMRKNRLVVTNLDKLHTALSELCFSINYVPN
MVVWEHTFTPREYLTSHLEIRFTKSIVGMTMYNQATQEIAKPSELLTSVRAYMTVLQSIENYVQIDITRVFNNVLLQQTQHLDSHGE
PTITSLYTNWYLETLLRQVSNGHIAYFPAMKAFVNLPTENELTFNAEEYSDISEMRSLSELLGPYGMKFLSESLMWHISSQVAELKK
LVVENVDVLTQMRTSFDKPDQMAALFKRLSSVDSVLKRMTIIGVILSFRSLAQEALRDVLSYHIPFLVSSIEDFKDHIPRETDMKVA
MNVYELSSAAGLPCEIDPALVVALSSQKSENISPEEEYKIACLLMVFVAVSLPTLASNVMSQYSPAIEGHCNNIHCLAKAINQIAAA
LFTIHKGSIEDRLKEFLALASSSLLKIGQETDKTTTRNRESVYLLLDMIVQESPFLTMDLLESCFPYVLLRNAYHAVYKQSVTSSA

**>WAVE1 (1-230)**
GHMPLVKRNIDPRHLCHTALPRGIKNELECVTNISLANIIRQLSSLSKYAEDIFGELFNEAHSFSFRVNSLQERVDRLSVSVTQLDP
KEEELSLQDITMRKAFRSSTIQDQQLFDRKTLPIPLQETYDVCEQPPPLNILTPYRDDGKEGLKFYTNPSYFFDLWKEKMLQDTEDK
RKEKRKQKQKNLDRPHEPEKVPRAPHDRRREWQKLAQGPELAEDDANLLHKHIEVANG

**>WAVE1 (1-230)-WCA,** or **WAVE1 (1-230)-(GGS)$_6$-WCA(485-559)**
GHMPLVKRNIDPRHLCHTALPRGIKNELECVTNISLANIIRQLSSLSKYAEDIFGELFNEAHSFSFRVNSLQERVDRLSVSVTQLDP
KEEELSLQDITMRKAFRSSTIQDQQLFDRKTLPIPLQETYDVCEQPPPLNILTPYRDDGKEGLKFYTNPSYFFDLWKEKMLQDTEDK
RKEKRKQKQKNLDRPHEPEKVPRAPHDRRREWQKLAQGPELAEDDANLLHKHIEVANG**GGSGGSGGSGGSGGSGGS**KRHPSTLPVIS
DARSVLLEAIRKGIQLRKVEEQREQEAKHERIENDVATILSRRIAVEYSDSEDDSEFDEVDWLE

**>WAVE1 (1-230)-Rac1,** or **WAVE1 (1-230)-(GGS)$_6$-Rac1$^{Q61L/P29S}$(1-188)**
GHMPLVKRNIDPRHLCHTALPRGIKNELECVTNISLANIIRQLSSLSKYAEDIFGELFNEAHSFSFRVNSLQERVDRLSVSVTQLDP
KEEELSLQDITMRKAFRSSTIQDQQLFDRKTLPIPLQETYDVCEQPPPLNILTPYRDDGKEGLKFYTNPSYFFDLWKEKMLQDTEDK
RKEKRKQKQKNLDRPHEPEKVPRAPHDRRREWQKLAQGPELAEDDANLLHKHIEVANG**GGSGGSGGSGGSGGSGGS**MQAIKCVVVGD
GAVGKTCLLISYTTNAF**S**GEYIPTVFDNYSANVMVDGKPVNLGLWDTAG**L**EDYDRLRPLSYPQTDVFLICFSLVSPASFENVRAKWY
PEVRHHCPNTPIILVGTKLDLRDDKDTIEKLKEKKLTPITYPQGLAMAKEIGAVKYLECSALTQRGLKTVFDEAIRAVLCPPPVKKR
KRK

**>WAVE1$^{\Delta PPP}$,** $^{131}$**PPPLNI**$^{136}$ replaced by **(GS)$_3$** in **WAVE1 (1-230)-WCA**
GHMPLVKRNIDPRHLCHTALPRGIKNELECVTNISLANIIRQLSSLSKYAEDIFGELFNEAHSFSFRVNSLQERVDRLSVSVTQLDP
KEEELSLQDITMRKAFRSSTIQDQQLFDRKTLPIPLQETYDVCEQ**GSGSGS**LTPYRDDGKEGLKFYTNPSYFFDLWKEKMLQDTEDK
RKEKRKQKQKNLDRPHEPEKVPRAPHDRRREWQKLAQGPELAEDDANLLHKHIEVANG**GGSGGSGGSGGSGGSGGS**KRHPSTLPVIS
DARSVLLEAIRKGIQLRKVEEQREQEAKHERIENDVATILSRRIAVEYSDSEDDSEFDEVDWLE

**>WAVE1$^{Y151E}$, Y151E in WAVE1 (1-230)-WCA**
GHMPLVKRNIDPRHLCHTALPRGIKNELECVTNISLANIIRQLSSLSKYAEDIFGELFNEAHSFSFRVNSLQERVDRLSVSVTQLDP
KEEELSLQDITMRKAFRSSTIQDQQLFDRKTLPIPLQETYDVCEQPPPLNILTPYRDDGKEGLKF**E**TNPSYFFDLWKEKMLQDTEDK
RKEKRKQKQKNLDRPHEPEKVPRAPHDRRREWQKLAQGPELAEDDANLLHKHIEVANG**GGSGGSGGSGGSGGSGGS**KRHPSTLPVIS
DARSVLLEAIRKGIQLRKVEEQREQEAKHERIENDVATILSRRIAVEYSDSEDDSEFDEVDWLE

**>Abi2 (1-158)**
GHMAELQMLLEEEIPGGRRALFDSYTNLERVADYCENNYIQSADKQRALEETKAYTTQSLASVAYLINTLANNVLQMLDIQASQLRR
MESSINHISQTVDIHKEKVARREIGILTTNKNTSRTHKIIAPANLERPVRYIRKPIDYTILDDIGHGVKVSTQ

**>HSPC300**
GHMGAAMAGQEDPVQREIHQDWANREYIEIITSSIKKIADFLNSFDMSCRSRLATLNEKLTALERRIEYIEARVTKGETLT

**>WCA**
KRHPSTLPVISDARSVLLEAIRKGIQLRKVEEQREQEAKHERIENDVATILSRRIAVEYSDSEDDSEFDEVDWLE

>GST-**Rac1$^{P29S}$** or GST-Tev-**Rac1$^{P29S}$(1-188)**
MSPILGYWKIKGLVQPTRLLLEYLEEKYEEHLYERDEGDKWRNKKFELGLEFPNLPYYIDGDVKLTQSMAIIRYIADKHNMLGGCPK
ERAEISMLEGAVLDIRYGVSRIAYSKDFETLKVDFLSKLPEMLKMFEDRLCHKTYLNGDHVTHPDFMLYDALDVVLYMDPMCLDAFP
KLVCFKKRIEAIPQIDKYLKSSKYIAWPLQGWQATFGGGDHPPKSDLVPRGSENLYFQGHMQAIKCVVVGDGAVGKTCLLISYTTNA
F**S**GEYIPTVFDNYSANVMVDGKPVNLGLWDTAGQEDYDRLRPLSYPQTDVFLICFSLVSPASFENVRAKWYPEVRHHCPNTPIILVG
TKLDLRDDKDTIEKLKEKKLTPITYPQGLAMAKEIGAVKYLECSALTQRGLKTVFDEAIRAVLCPPPVKKRKRK

>GST-**Rac1$^{QP}$** or GST-Tev-**Rac1$^{Q61L/P29S}$(1-188)**
MSPILGYWKIKGLVQPTRLLLEYLEEKYEEHLYERDEGDKWRNKKFELGLEFPNLPYYIDGDVKLTQSMAIIRYIADKHNMLGGCPK
ERAEISMLEGAVLDIRYGVSRIAYSKDFETLKVDFLSKLPEMLKMFEDRLCHKTYLNGDHVTHPDFMLYDALDVVLYMDPMCLDAFP
KLVCFKKRIEAIPQIDKYLKSSKYIAWPLQGWQATFGGGDHPPKSDLVPRGSENLYFQGHMQAIKCVVVGDGAVGKTCLLISYTTNA
F**S**GEYIPTVFDNYSANVMVDGKPVNLGLWDTAG**L**EDYDRLRPLSYPQTDVFLICFSLVSPASFENVRAKWYPEVRHHCPNTPIILVG
TKLDLRDDKDTIEKLKEKKLTPITYPQGLAMAKEIGAVKYLECSALTQRGLKTVFDEAIRAVLCPPPVKKRKRK

**Supplementary Table 3. Cryo-EM data collection, refinement, and validation statistics.**

| Sample name | **WRC**$^{apo}$ | **WRC**$^{D\text{-}Rac1}$ | **WRC**$^{AD\text{-}Rac1}$ |
|---|---|---|---|
| EMDB ID | EMD-26732 | EMD-26733 | EMD-26734 |
| PDB ID | 7USC | 7USD | 7USE |
| | | | |
| Microscope | Talos Arctica | Talos Arctica | Talos Arctica |
| Detector (Mode) | Falcon 3EC counting mode | Falcon 3EC counting mode | Falcon 3EC counting mode |
| Voltage (kV) | 200 | 200 | 200 |
| Magnification (nominal) | 120,000 | 120,000 | 120,000 |
| Total electron fluence ($e^-$/Å$^2$) | 44.06 | 45.27 | 41.34 |
| Electron flux ($e^-$/pixel/sec) | 0.84 | 0.87 | 0.79 |
| Defocus range (μm) | -0.6 to -1.2 | -0.5 to -1.0 | -0.8 to -1.2 |
| Pixel size (Å) | 0.8757 | 0.8757 | 0.8757 |
| Total exposure time (sec) | 40 | 40 | 40 |
| Total fractions/micrograph | 62 | 62 | 62 |
| Exposure per fraction ($e^-$/Å$^2$/frame) | 0.71 | 0.73 | 0.67 |
| Micrographs collected (no.) | 2913 | 2512 | 1285 |
| Total extracted particles (no.) | 2,006,821 | 1,765,193 | 666,417 |
| Particles used for 3D analyses (no.) | 957,365 | 856,797 | 657,065 |
| Final refined particles (no.) | 95,319 | 87,810 | 139,296 |
| Symmetry imposed | C1 | C1 | C1 |
| FSC 0.5 (masked/unmasked)<br>FSC 0.143 (masked/unmasked) | 3.5/4.1<br>3.0/3.4 | 3.5/4.2<br>3.0/3.5 | 3.6/4.0<br>3.0/3.3 |
| FSC Sphericity | 0.925 | 0.911 | 0.884 |
| Local resolution range (Å) | 2.8 – 4.5 | 2.8 – 4.5 | 2.5 – 4.5 |
| Map Sharpening *B* factors (Å$^2$) | -35 | -39 | -59 |
| | | | |
| **Model composition** | | | |
| Non-hydrogen atoms | 21709 | 23226 | 23913 |
| Protein residues | 2673 | 2864 | 2954 |
| Ligands | 0 | 2 | 4 |
| **Refinement** | | | |
| Refinement package (s) | Phenix | Phenix | Phenix |
| Map Correlation Coefficient | | | |
| Global | 0.86 | 0.80 | 0.78 |
| Local | 0.87 | 0.81 | 0.78 |
| R.m.s. deviations | | | |
| Bond lengths (Å) | 0.007 | 0.005 | 0.006 |
| Bond angles (°) | 0.992 | 0.990 | 1.058 |
| **Validation** | | | |
| EMRinger score | 2.84 | 2.15 | 2.35 |
| MolProbity score | 1.53 | 1.39 | 1.35 |
| Clashscore | 6.44 | 7.13 | 6.41 |
| Poor rotamers (%) | 0 | 0.15 | 0.19 |
| Cβ deviations (%) | 0 | 0.07 | 0 |
| Ramachandran plot | | | |
| Favored (%) | 97.01 | 98.23 | 98.46 |
| Allowed (%) | 2.99 | 1.77 | 1.54 |
| Disallowed (%) | 0.00 | 0.00 | 0.00 |
| CaBLAM outliers (%) | 0.73 | 0.64 | 0.76 |

## Supplementary Table 4. DNA oligos used in this study

| Purpose | Identifier and sequence, all 5'-3' |
|---|---|
| Sra1$^{D\text{-}Rac1}$ | yso-071316-1, GGTGGATCAGGCGGGTCG, Rac SLIC from WAVE1(1-230)-(GGS)6-Rac1 QP dC4 for Sra CT-(GGS)4-Rac-D site fw<br>yso-071316-2, GATCCTCTAGTACTTCTCGACAAGCTTTCATTTTCTCTTCCTCTTCTTCACGGGAG, Rac SLIC for Sra CT-(GGS)4-Rac-D site bw<br>yso-071316-3, TGAAAGCTTGTCGAGAAGTACTAGAG, Sra1 SLIC for Sra CT-(GGS)4-Rac-D site fw<br>yso-071316-4, GCCAGACCCACCCGACCCGCCTGATCCACCGCTGCTGGCGAGGGACTGG, Sra1 SLIC for Sra CT-(GGS)4-Rac-D site bw |
| Sra1$^{D\text{-}Rac1}$ | yso-071316-5, GGTGGCTCTGGAGGGTCC, WAVE1-(GGS)6-Rac AliBlunt fw<br>yso-071316-17, CCCGCTACCCGATCCGCTGCCTGACCCAGAACCTTTTCTCTTCCTCTTCTTCACGGGAG, SLIC Rac into Sra1 loops bw<br>yso-071316-18, TCTGGGTCAGGCAGCGGATCGGGTAGCGGGAGTTCCAACAAGGACTGCCCCGAC, SLIC to insert Rac into Sra1 Y423 loop fw<br>yso-071316-19, GCCTGATCCACCGGACCCTCCAGAGCCACCGTACTTGTCGGTGGGGTGCAC, SLIC to insert Rac into Sra1 Y423 loop bw |
| Sra1$^{AD\text{-}Rac1}$ | yso-071316-3, TGAAAGCTTGTCGAGAAGTACTAGAG, Sra1 SLIC for Sra CT-(GGS)4-Rac-D site fw<br>cbyo-200701-2, GCTGCTGGCGAGGGACTGG, Sra1 SLIC for Sra CT-(GGS)4-Rac-D site bw |
| Sra1$^{N183R}$ | Yso-210311-5, GCGACCACTCAGCGTACAAGAG, PCR to make N183R fw<br>Yso-210311-6, GCTTCACACTGCACTTCATGTTCTTC, PCR to make N183R bw |
| Sra1$^{S186M}$ | Yso-210311-7, TGGCGTACAAGAGGGCCGCTC, PCR to make S186M fw<br>Yso-210311-8, TGTGGTCGTTCTTCACACTGCACTTC, PCR to make S186M bw |
| Sra1$^{K189M}$ | Yso-210311-9, TGAGGGCCGCTCAGTTTTTAC, PCR to make K189M fw<br>Yso-210311-10, TGTACGCTGAGTGGTCGTTC, PCR to make K189M bw |
| Sra1$^{Y108A}$ | Yso-210311-1, CCGAGAAAACCGTGGAGGTTCTG, PCR to make Y108A fw<br>Yso-210311-2, CGATTTCCACTCTGTTAGGCTGC, PCR to make Y108A bw |
| Sra1$^{Y108H}$ | Yso-210322-1, GAAATCCACGAGAAAACCGTGGAGGTTCTGGAGCCTGAG, PCR to make Y108H fw<br>Yso-210322-2, CGGTTTTCTCGTGGATTTCCACTCTGTTAGGCTGCTCGTTAC, PCR to make Y108H bw |
| Sra1$^{N176W}$ | Yso-210311-3, GGATGAAGTGCAGTGTGAAGAACG, PCR to make N176W fw<br>Yso-210311-4, ACTTCAGCTCGTCCAGCACAG, PCR to make N176W bw |
| Sra1$^{R87C,\ D\text{-}Rac1}$ | Yso-210311-36, CAGCTGCTCCCGGGCCATC, PCR to make R87C fw<br>Yso-210311-37, CACCAGGTGTACAGCATGACAGCATATTC, PCR to make R87C bw |
| Sra1 sequencing primers | cbyo-081210-1, TTCATACCGTCCCACCATC, fwd seq pAV5a MCS<br>cbyo-090407-1, GTTTCAGGTTCAGGGGGAGGTG, pAV5a sequencing bw<br>cbyo-150731-1, GTGAAGAACGACCACTCAGC, sequencing hSra1 new-mid1<br>cbyo-150731-2, ATTCCTGGAAGCTTGTGCAC, sequencing hSra1 new-mid2<br>cbyo-150731-3, CACATCCTGGAGACCAAGG, sequencing hSra1 new-mid3<br>cbyo-150731-4, TGCACAGCCTCAGTATCTGC, sequencing hSra1 new-mid4 |
| WAVE1$^{\Delta PPP}$ | Ylo-210705-1, TCCGGCTCTCTCACTCCTTATAGAGATGATGGTAAAGAAG, Aliblunt for WAVE230VCA ΔPPP fw<br>Ylo-210705-2, TCCAGAACCCTGTTCACAAACATCGTACGTCTCC, Aliblunt for WAVE230VCA ΔPPP bw |
| WAVE1$^{Y151E}$ | Yso-210311-27, AGACCAATCCTTCGTATTTCTTTGATC, PCR to make MBP-WAVE1 1-230 VCA Y151E fw<br>Yso-210311-28, CAAACTTCAGACCTTCTTTACCATC, PCR to make MBP-WAVE1 1-230 VCA Y151E bw |
| EGFP-mCyfip1$^{Y108A}$ | CYFIP1_Y108A_fwd, CGAATAGAGTTGAAATTGCTGAGAAAACCGTGG<br>CYFIP1_Y108A_rev, CCACGGTTTTCTCAGCAATTTCAACTCTATTCG |
| EGFP-mCyfip1$^{N176W}$ | CYFIP1_N176W_fwd, GGATGAGCTGAAGTGGATGAAGTGCAGTG<br>CYFIP1_N176W_rev, CACTGCACTTCATCCACTTCAGCTCATCC |
| EGFP-mCyfip1$^{N183R}$ | CYFIP1_N183R_fwd, GTGCAGTGTGAAGAGAGACCACTCTGC<br>CYFIP1_N183R_rev, GCAGAGTGGTCTCTCTTCACACTGCAC |
| EGFP-mCyfip1$^{S186M}$ | CYFIP1_S186M_fwd, GTGAAGAATGACCACATGGCATATAAGAGGG<br>CYFIP1_S186M_rev, CCCTCTTATATGCCATGTGGTCATTCTTCAC |
| EGFP-mCyfip1$^{K189M}$ | CYFIP1_K189M_fwd, GACCACTCTGCATATATGAGGGCTGCTCAG<br>CYFIP1_K189M_rev, CTGAGCAGCCCTCATATATGCAGAGTGGTC |
| mCyfip1 sequencing primers | CYFIP1-498-Seq-r, TCATCCAACACAGCAAACATG<br>CYFIP1-353-Seq-fwd, TGGAAGTCCTTGAACCCG<br>CYFIP1-981-Seq-f, CAGCGCACACTATGAGGAG<br>CYFIP1-2498-Seq-r, GGTAATTCTTCCATAAGGTGCAG<br>CYFIP1-2401-Seq-f, ACATCAGTAGTCGAGCTAGATGGA<br>CYFIP1-3100-Seq-f, CCTTTCCAGAATATCTTACCTCGA |

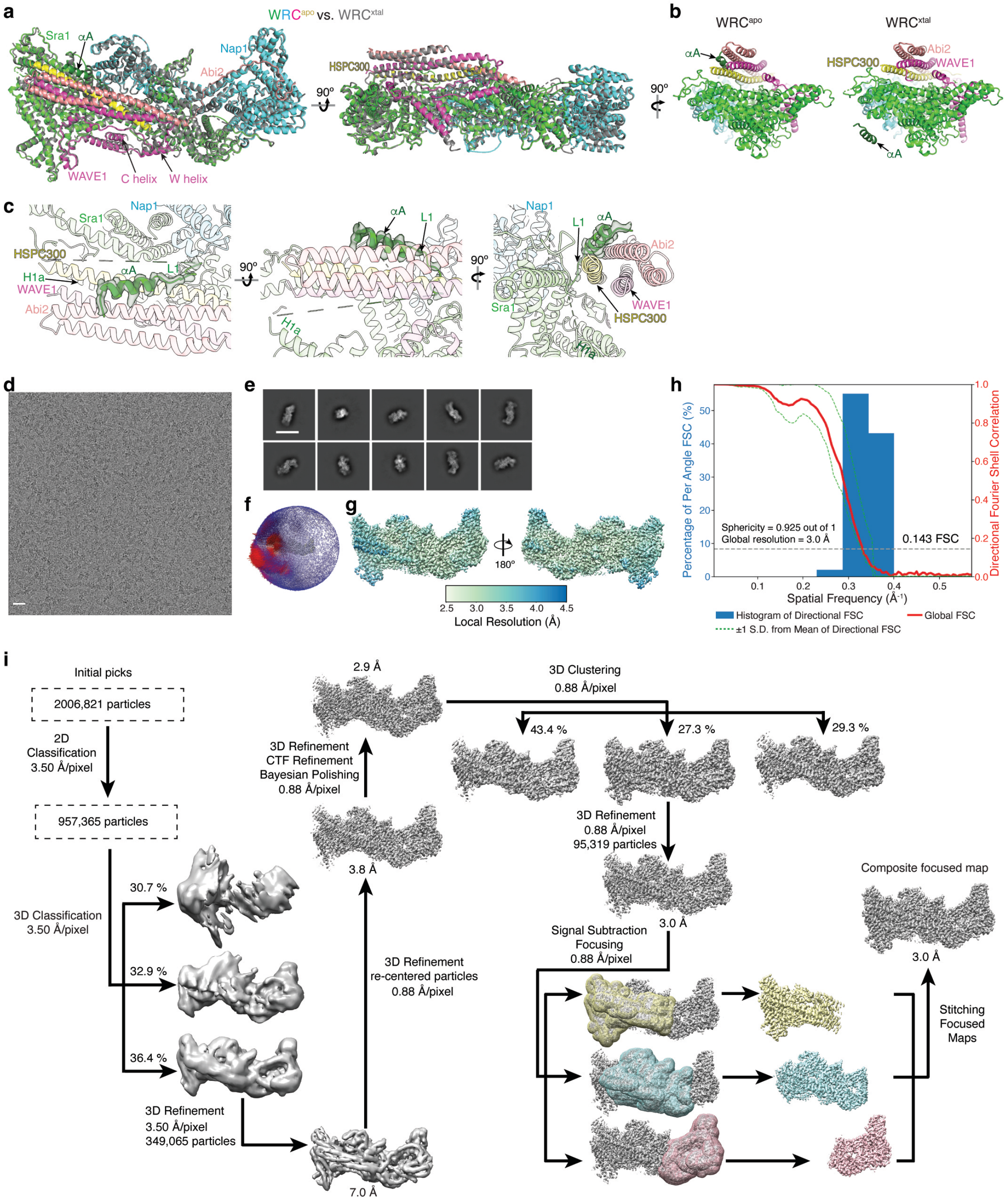


**Supplementary Fig. 1 Determination of WRC$^{apo}$ structure by cryo-EM. (a)** Structural overlay of WRC$^{apo}$ (color) and WRC$^{xtal}$ (grey, PDB: 3P8C), showing high similarity between the two structures, with the whole complex r.m.s.d. = 0.827 Å as calculated in Pymol. **(b)** Side-by-side comparison of WRC$^{apo}$ and WRC$^{xtal}$ showing the difference in the assigned position of the αA helix. In WRC$^{xtal}$ structure, this helix was assigned to a neighboring WRC in the crystal lattice, which raised the hypothesis that this helix might

promote WRC clustering or oligomerization at membranes. By contrast, the single particle cryo-EM structure of $WRC^{apo}$ reveals the helix belongs to the same WRC. **(c)** Density for the αA helix and L1 loop (a.a. 27-56) in Sra1. The weak density of the L1 loop following the C-terminus of αA suggests L1 must wind through an internal cavity in WRC to connect to the N-terminus of the H1a helix. This buried loop should restrain αA from approaching another WRC, unless the complex is first disassembled. The missing density of the C-terminal half of the L1 loop is indicated by a dashed line connecting to the N-terminus of the H1a helix. **(d)** A representative cryo-EM micrograph of vitrified $WRC^{apo}$ sample, from a data set comprising 2,913 micrographs. Scale bar: 20 nm. **(e)** Representative 2D class averages of $WRC^{apo}$. Scale bar: 20 nm. **(f)** Plot showing the Euler angle distribution assigned to the particles contributing to the final reconstructed map of $WRC^{apo}$. The height of each cylinder corresponds to the number of particles in each angular orientation. **(g)** Maps of $WRC^{apo}$ colored based on local resolution values and showing two views that are rotated 180° along y-axis. **(h)** Directional Fourier Shell Correlation (FSC) plot representing 3D resolution anisotropy in the cryo-EM map of $WRC^{apo}$. The blue histograms represent percentage of directional resolution over the spatial frequency; the red line indicates the global FSC; the green dashed lines correspond to ±1 standard deviation from mean of directional resolutions; and the grey dashed line shows FSC at the cut-off value 0.143. **(i)** Schematic showing cryo-EM data processing steps for obtaining 3D reconstruction of $WRC^{apo}$ complex dataset. ~2 million particles went through multiple iterations of 2D classification and one round of 3D classification to clean up the particle stack. 3D clustering helped to further sort out heterogeneity existing in the data set. This clean stack of particles was subject to 3D auto-refinement and signal-subtracted focused refinement. The focused maps were combined to generate the final composite map.

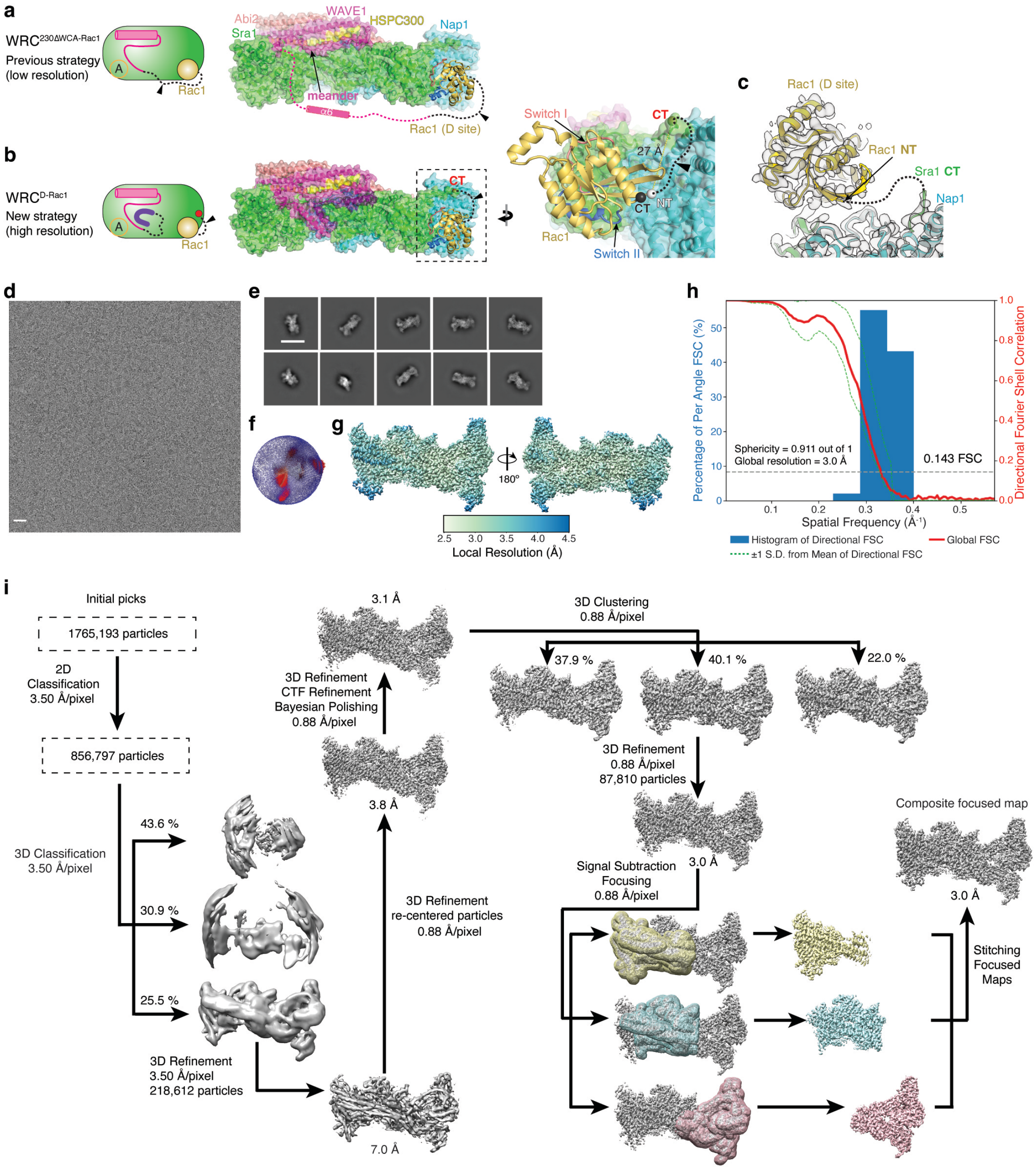


**Supplementary Fig. 2 Cryo-EM structure determination of WRC$^{D\text{-}Rac1}$. (a-b)** Cartoon and structural representations to show the previous and new strategies of stabilizing Rac1 binding to the D site. Dotted lines and arrow heads indicate flexible (GGS) or (GS) linkers (see **Supplementary Table 1 & 2** for linker details). **(c)** Density for Rac1 tethered to the D site, showing no observable density accountable for the flexible linker used for tethering (indicated by dotted line). The WRC$^{D\text{-}Rac1}$ structure is overlaid on WRC$^{apo}$ (pale color), showing the local structures surrounding the tethering points were not perturbed. **(d)** A

representative cryo-EM micrograph of vitrified $WRC^{D\text{-}Rac1}$ sample, from a data set of 2,512 micrographs. Scale bar: 20 nm. **(e)** Representative 2D class averages of $WRC^{D\text{-}Rac1}$. Scale bar: 20 nm. **(f)** Plot showing the Euler angle distribution of particles that contributed to final reconstruction of $WRC^{D\text{-}Rac1}$. The height of each cylinder corresponds to the number of particles in each angular orientation. **(g)** Maps of $WRC^{D\text{-}Rac1}$ colored based on local resolution values and showing two views rotated by 180° along y-axis. **(h)** Directional Fourier Shell Correlation (FSC) plot representing 3D resolution anisotropy in the cryo-EM map of $WRC^{D\text{-}Rac1}$. The blue histograms represent percentage of directional resolution over the spatial frequency; the red line indicates the global FSC; the green dashed lines correspond to ±1 standard deviation from mean of directional resolutions; and the grey dashed line shows FSC at the cut-off value 0.143. **(i)** A schematic of the different data processing steps of $WRC^{D\text{-}Rac1}$ complex dataset, which is similar to that of $WRC^{apo}$.

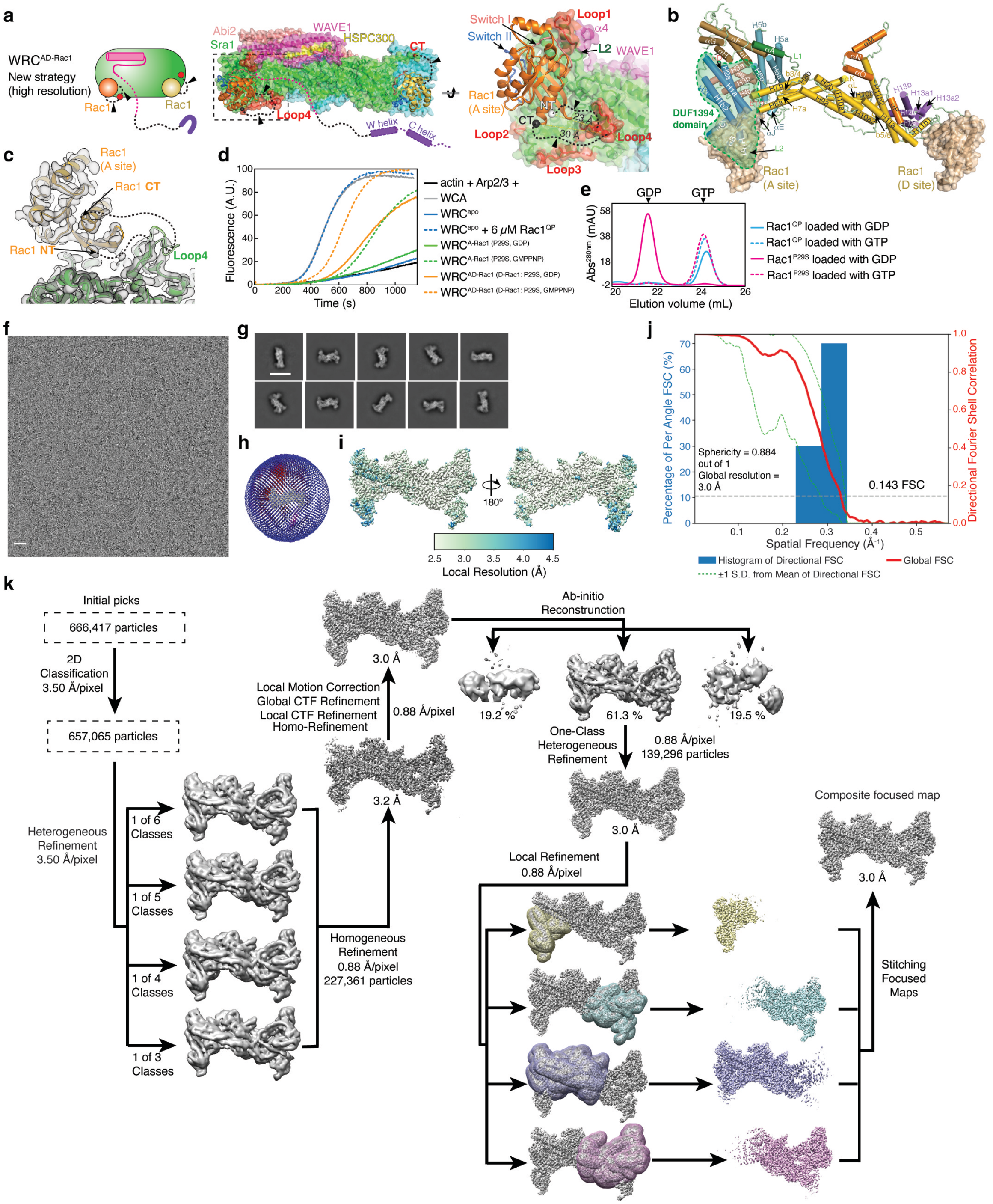


**Supplementary Fig. 3 Cryo-EM structure determination of WRC$^{AD\text{-}Rac1}$.** **(a)** Cartoon and structural representation of the new strategies to stabilize Rac1 binding to the A site. Dotted lines and arrow heads indicate flexible (GGS) or (GS) linkers (see **Supplementary Table 1 & 2** for linker details). Four non-conserved surface loops in Sra1 (red, Loop1: a.a. 95-103; Loop2: a.a. 276-281; Loop3: a.a. 329-342; Loop4: a.a. 418-432) surrounding the

A site were chosen to insert Rac1 using two separate flexible linkers. Insertion at Loop4 between Y423 and S424 produced the high-resolution cryo-EM structure of $WRC^{AD\text{-}Rac1}$. **(b)** Secondary structure assignment of Sra1, following the same scheme used in the crystal structure of the WRC[5]. DUF1394 domain and Rac1 at both A and D site are indicated. **(c)** Density for Rac1 tethered to the A site, showing very weak, if any, density could be observed for the flexible linkers used for the tethering (indicated by dotted lines). The $WRC^{AD\text{-}Rac1}$ structure is overlaid on $WRC^{apo}$ (pale color), showing the local structures surrounding the tethering points were not disturbed, except that no density was observed for a.a. 422-427, which is the tip of Loop4 where Rac1 is inserted between a.a. 423 and 424. **(d)** Pyrene-actin polymerization assay showing tethering Rac1 to the A site was sufficient to promote WRC activation in a nucleotide dependent manner, and this activity was further promoted by Rac1 binding to the D site. The green curves show $WRC^{A\text{-}Rac1}$, in which Rac1 only contained the P29S mutation, instead of both P29S and Q61L. Unlike $Rac1^{QP}$, $Rac1^{P29S}$ can be loaded with GMPPNP or GDP, as is shown in **(e)**. The orange curves compare the activities of $WRC^{AD\text{-}Rac1}$, in which the D-site $Rac1^{P29S}$ was loaded with indicated nucleotides, while the A-site $Rac1^{QP}$ remained bound to GTP. Reactions use the KMEI20GD buffer (see Methods) and contain 4 $\mu$M actin (5% pyrene-labeled), 10 nM Arp2/3 complex, 100 nM WRC230WCA or WAVE1 WCA. **(e)** Ion exchange chromatography to identify the nucleotide bound to Rac1 after the loading procedures (see Methods for details), showing $Rac1^{P29S}$ can be loaded with GTP (or GMPPNP) and GDP, while $Rac1^{QP}$ stays bound to GTP after the same treatment. The bound nucleotides were released from Rac1 after urea denaturation, separated from the protein through a 3-kDa MWCO membrane, and analyzed by anion exchange chromatography. **(f)** A representative cryo-EM micrograph of vitrified $WRC^{AD\text{-}Rac1}$, from a data set comprising 1,285 micrographs. Scale bar: 20 nm. **(g)** Representative 2D class averages of $WRC^{AD\text{-}Rac1}$. Scale bar: 20 nm. **(h)** Plot showing the Euler angle distribution of the particles that contributed to the final reconstruction of $WRC^{AD\text{-}Rac1}$. The height of each cylinder corresponds to the number of particles in each angular orientation. **(i)** Maps of $WRC^{AD\text{-}Rac1}$ colored based on local resolution values and showing two different views that are rotated 180° relative to y-axis. **(j)** Directional Fourier Shell Correlation (FSC) plot representing 3D resolution anisotropy in the cryo-EM map of $WRC^{AD\text{-}Rac1}$. The blue histograms represent percentage

of directional resolution over the spatial frequency; the red line indicates the global FSC; the green dashed lines correspond to ±1 standard deviation from mean of directional resolutions; and the grey dashed line shows FSC at the cut-off value 0.143. **(k)** A schematic for the different data processing steps for the WRC$^{AD\text{-}Rac1}$ dataset. Source data for **(d-e)** are provided as a Source Data file.

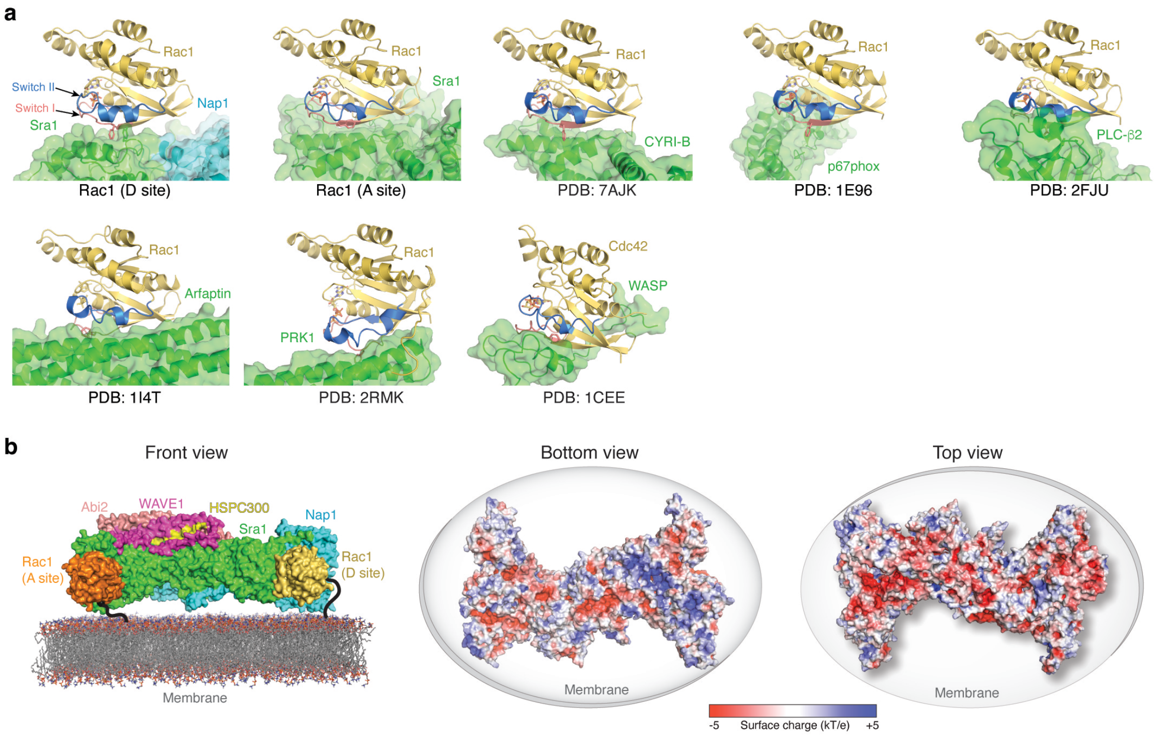


**Supplementary Fig. 4 Orientation of Rac1 binding to the WRC and other ligands. (a)** Comparison of Rac1 and Cdc42 binding to the indicated ligands, with GTPases remaining in the same orientation. **(b)** Surface representation (left) and surface charge representation (right, calculated using APBS in Pymol[6]) showing how WRC can be oriented on the membrane by binding to two Rac1 molecules and through electrostatic interactions between its positively charged surface (Bottom view) and acidic phospholipids on the membrane. Rac1 molecules are anchored on the membrane through prenylation of their C-terminal tails (indicated by black lines, ~15-30 Å in distance each).

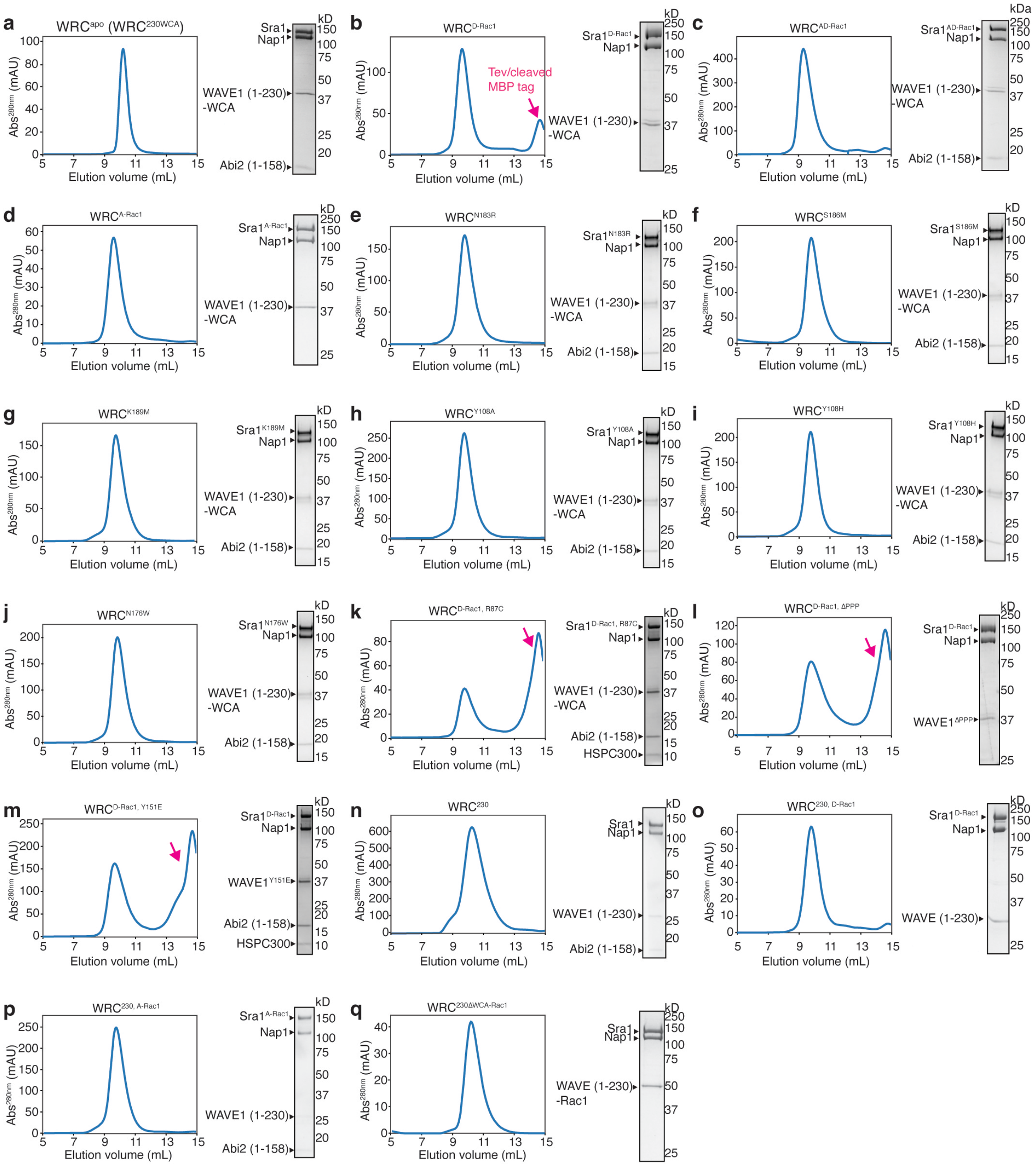


**Supplementary Fig. 5** Gel filtration and SDS-PAGE of purified WRCs used in this study. Shown are the final steps or analytical steps of WRC purification using a 24-ml Superdex 200 gel filtration column, with the Coomassie-blue stained SDS-PAGE gels showing the peak or pooled fractions. Depending on whether the preceding purification step includes a Source Q15 ion exchange column, different amounts of Tev and cleaved MBP tag may show as peaks (indicated by magenta arrows) that were well separated from the WRC peak.

Source data are provided as a Source Data file. Uncropped gel images are also shown at the bottom of this file.

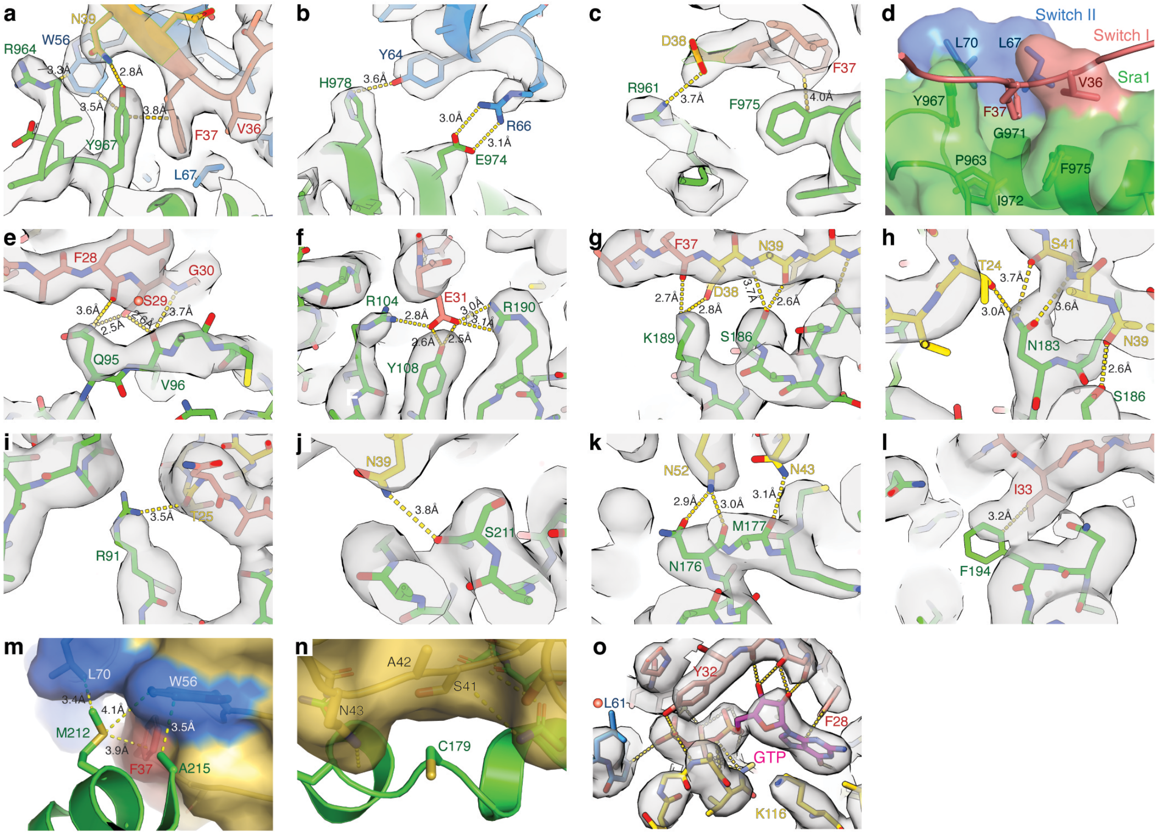


**p**

**A site**

| | Rac1 | Sra1 | distance (Å) |
|---|---|---|---|
| α1 | T24 | N183 | 3.0 |
| | T25 CO | R91 | 3.5 |
| Switch I | F28 CO | Q95 | 3.6 |
| | P29S | Q95 | 2.5 |
| | P29S | V96 CO | 2.6 |
| | G30 NH | V96 CO | 3.7 |
| | E31 | R104 | 2.8 |
| | E31 | Y108 | 2.5, 2.7 |
| | E31 | R190 | 3.0, 3.1 |
| | I33 | F194 | 3.2 |
| | F37 | M212 | 3.9 |
| | F37 CO | K189 | 2.7 |
| β2/β3 | D38 | K189 | 2.8 |
| | N39 NH | S186 | 3.7 |
| | N39 | S211CO | 3.8 |
| | N39 CO | S186 | 2.6 |
| | S41 NT | N183 | 3.6 |
| | S41 CO | N183 | 3.7 |
| | A42 | M632 | 4.7 |
| | N43 | M177 CO | 3.1 |
| | V44 | M632 | 4.5 |
| | N52 | N176 | 2.9 |
| | N52 | N176 CO | 3.0 |
| Switch II | W56 | M212 | 4.1 |
| | W56 | A215 | 3.5 |
| | L70 | M212 | 3.4 |

**D site**

| | Rac1 | Sra1 | distance (Å) |
|---|---|---|---|
| Switch I | V36 | F975 | 3.8 |
| | F37 | F975 | 4.0 |
| | F37 | Y967 | 3.8 |
| β2/β3 | D38 | R961 | 3.7 |
| | N39 | Y967 | 2.8 |
| Switch II | W56 | R964 | 3.3 |
| | W56 | Y967 | 3.5 |
| | Y64 | H978 | 3.6 |
| | R66 | E974 | 3.0, 3.1 |

**CYRI-B**

| | Rac1 | CYRI-B | distance (Å) |
|---|---|---|---|
| α1 | T25 | N154 | 3.5 |
| | T25 CO | R64 | 3.2 |
| Switch I | N26 CO | R64 | 3.1 |
| | F28 NH | Q68 | 3.6 |
| | F37 CO | R160 | 2.9 |
| | F37 | R160 | 2.9 |
| | F37 | L189 | 4.5 |
| β2/β3 | D38 | R160 | 3.3 |
| | N39 | Q153 | 3.4 |
| | N39 CO | S157 | 2.6 |
| | N39 | S157 | 3.8 |
| | N39 | S188 CO | 2.5 |
| | N39 | S188 | 3.8 |
| | N39 | A192 NH | 3.3 |
| | S41 | Q153 | 3.1 |
| | N52 | M147 CO | 3.5 |
| Switch II | W56 | L189 | 4.0 |
| | W56 | A192 | 3.5 |
| | W56 | A192 CO | 3.7 |
| | L70 | L189 | 3.7 |

- Hydrogen bonds & weak polar interactions
- Salt bridges
- Hydrophobic interactions

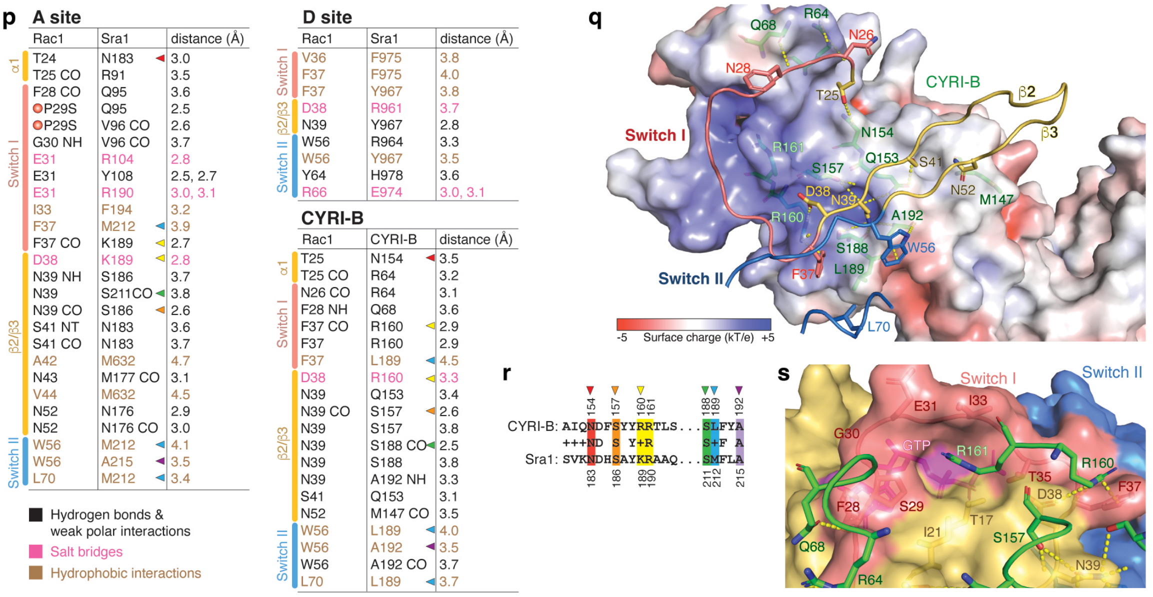


**Supplementary Fig. 6 Details of interactions at A and D sites and comparison with CYRI-B. (a-o)** Detailed views of map density or semitransparent surface presentation of key residues that mediate Rac1 binding to the D site **(a-d)** and A site **(e-n)**, and GTP bound to Rac1$^{QP}$ at the A site **(o)**. Red dots indicated the P29S or Q61L mutation in Rac1 used for optimizing A site binding. In **(n)**, C179 in Sra1 does not have specific interactions with

Rac1, but is packed against a concave pocket on Rac1. The structure suggests mutating C179, which is limited to small side chains throughout all examined organisms, to the long-chain residue Arginine (C179R) would cause steric clashes to disrupt Rac1 binding and WRC activation. **(p)** Contacting residues between Rac1 and indicated surface. Color-coded arrow heads indicate conserved interactions shared by both Sra1 and CYRI-B. **(q)** Top view and semitransparent surface charge representation of Rac1 binding to the CYRI-B surface (PDB: 7AJK). Yellow dotted lines indicate polar interactions. For clarity, the backbone of Rac1 Switch I-β2-β3-Switch II sequence mediating the binding is shown as loops. Orientation is similar to Fig. 3B. **(r)** Sequence alignment of the conserved residues of CYRI-B and Sra1 mediating Rac1 binding, which are indicated by color arrowheads also shown in (P). **(s)** Semitransparent surface representation of the Rac1 surface, showing how $CYRI^{R161}$ fits into the pocket in Rac1 similar to $Sra1^{R190}$ shown in **Fig. 3c**.

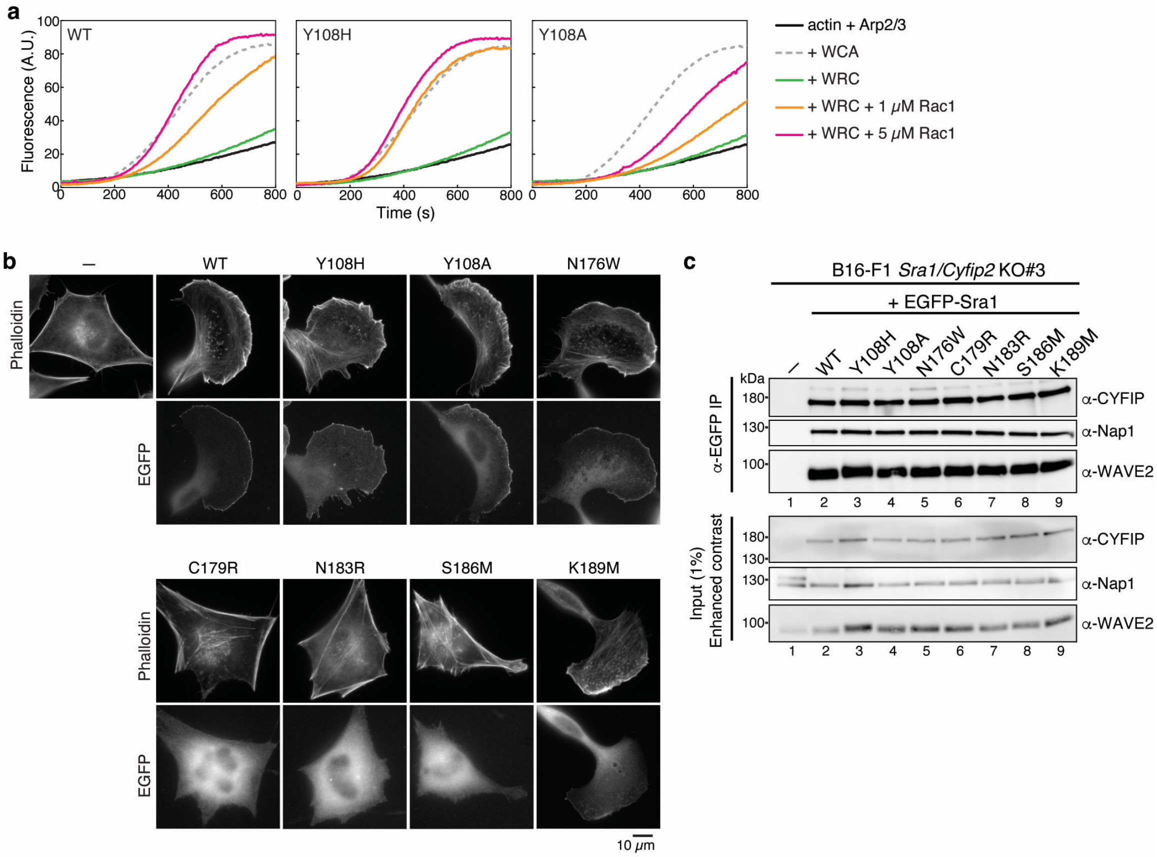


**Supplementary Fig. 7 Additional information of Sra1 A site mutations in Fig. 3. (a)** Pyrene-actin polymerization assays comparing the activities of WRCs carrying Y108H *vs*. Y108A. Reactions use the NMEH20GD buffer (see Methods) and contain 3.5 $\mu$M actin (5% pyrene-labeled), 10 nM Arp2/3 complex, 100 nM WRC230WCA or WAVE1 WCA, and/or indicated amounts of untagged Rac1$^{QP}$. **(b)** Representative fluorescence images of B16-F1 *Sra1*/*Cyfip2* double KO#3 cells transfected with indicated EGFP-Sra1 variants, stained by phalloidin for F-actin, and imaged for both actin and EGFP-Sra1. Data is representative of 3 independent repeats. **(c)** Immunoprecipitation (IP) and Western blot of the same B16-F1 *Sra1*/*Cyfip2* double KO#3 cells used in **(b)**, which were transfected with indicated EGFP-tagged Sra1 variants, lysed, and probed for the expression and assembly of the WRC, as exemplified by CYFIP antibodies (α-CYFIP, which detected both Sra1 and Cyfip2), α-Nap1, and α-WAVE2. The experiment was performed once. Source data for **(a)** are provided as a Source Data file. Uncropped images for **(c)** are shown at the bottom of this file.

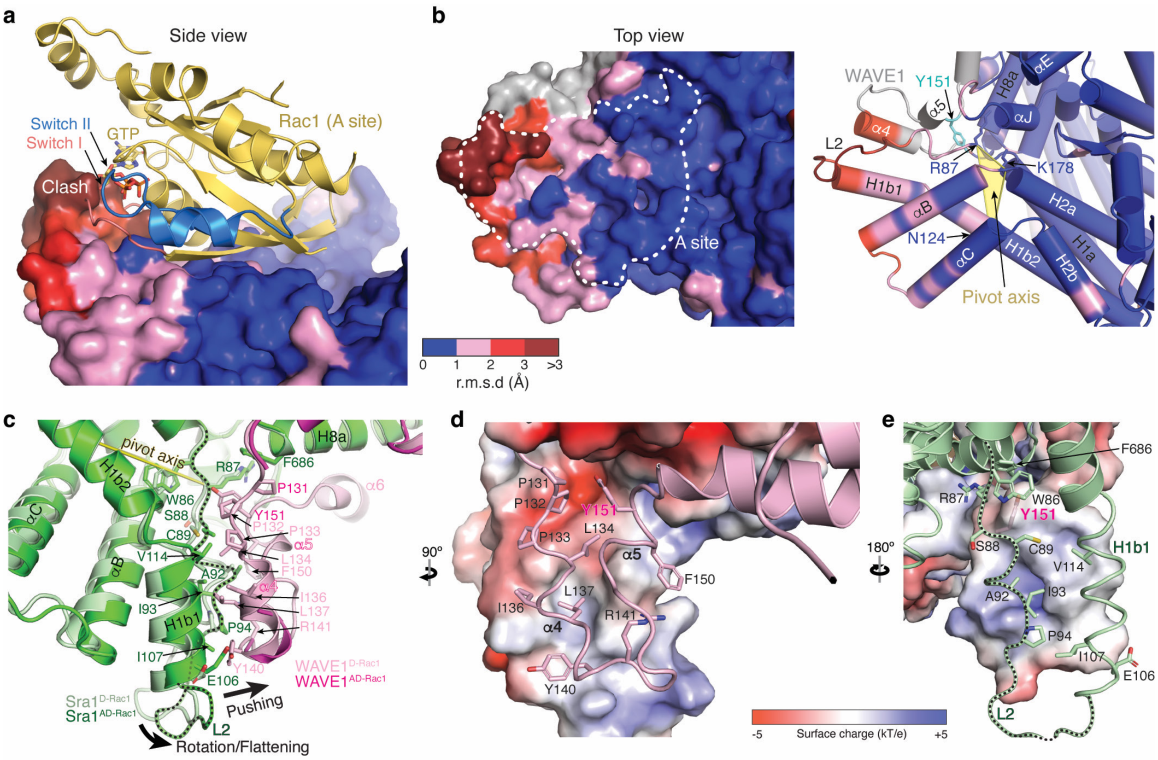


**Supplementary Fig. 8 Interactions propagating from A site binding to WCA release.** **(a)** Side view of the surface representation of the A site in WRC$^{\text{D-Rac1}}$, colored by r.m.s.d. values between WRC$^{\text{D-Rac1}}$ and WRC$^{\text{AD-Rac1}}$ using the *colorbyrmsd.py* script written by Shivender Shandilya, Jason Vertrees, and Thomas Holder (*https://pymolwiki.org/index.php/ColorByRMSD*). The two overall structures are aligned by excluding the regions that undergo major conformational changes (a.a. 56-337 of Sra1 and 131-544 of WAVE1). The A site binding Rac1 in WRC$^{\text{AD-Rac1}}$ is shown in cartoon to demonstrate the steric clash with the A site in WRC$^{\text{D-Rac1}}$. **(b)** Top view of the A site in surface (left) and cartoon (right) representations, following the same r.m.s.d. color scheme used in **(a)**. The grey color corresponds to the WAVE1 sequence released in WRC$^{\text{AD-Rac1}}$ (including Y151 in cyan). White dashed line indicates the boundary of the A site. Pivot axis for A site rotation/flattening is defined by a plane in yellow that runs through R87/N124/K178 and aligns to Y151 in WAVE1. **(c)** Detailed view of the conformational changes at the interface between H1b1-L2 of Sra1 and α4-loop-α5 of WAVE1. WRC$^{\text{D-Rac1}}$ structure is in light color. WRC$^{\text{AD-Rac1}}$ is in dark color. Black dotted line traces the L2 loop. Contacting residues are shown as sticks. **(d-e)** Electrostatic surface representations of the binding surfaces on the Sra1 side **(d)** and the WAVE1 side **(e)**.

**References for supplementary information**

1. Ismail, A. M., Padrick, S. B., Chen, B., Umetani, J. & Rosen, M. K. The WAVE Regulatory Complex is Inhibited. *Nat. Struct. Mol. Biol.* **16**, 561–563 (2009).
2. Chen, B. *et al.* Rac1 GTPase activates the WAVE regulatory complex through two distinct binding sites. *Elife* **6**, e29795 (2017).
3. Schaks, M. *et al.* Distinct Interaction Sites of Rac GTPase with WAVE Regulatory Complex Have Non-redundant Functions in Vivo. *Curr. Biol.* **28**, (2018).
4. Schaks, M., Reinke, M., Witke, W. & Rottner, K. Molecular Dissection of Neurodevelopmental Disorder-Causing Mutations in CYFIP2. *Cells* **9**, (2020).
5. Chen, Z. *et al.* Structure and Control of the Actin Regulatory WAVE Complex. *Nature* **468**, 533–538 (2010).
6. Jurrus, E. *et al.* Improvements to the APBS biomolecular solvation software suite. *Protein Sci.* **27**, (2018).

## Uncropped gel images for Supplementary Figure 5

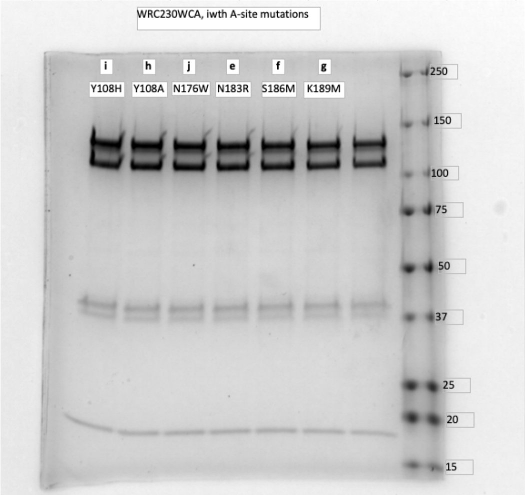


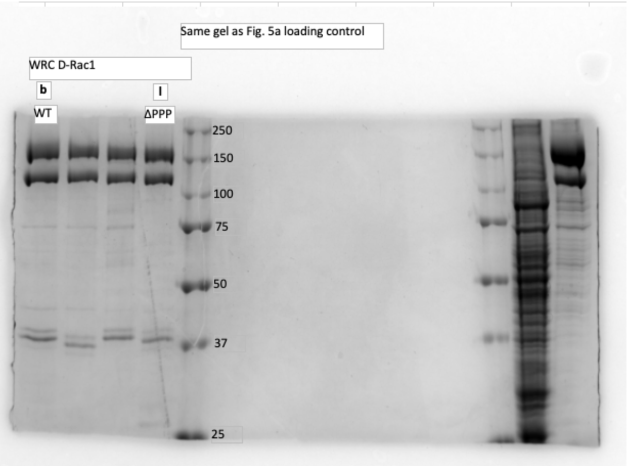


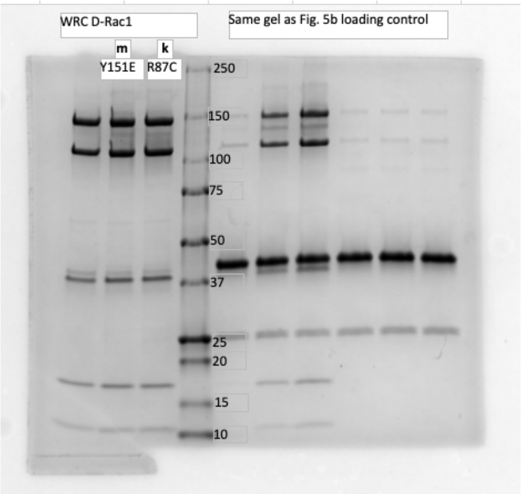


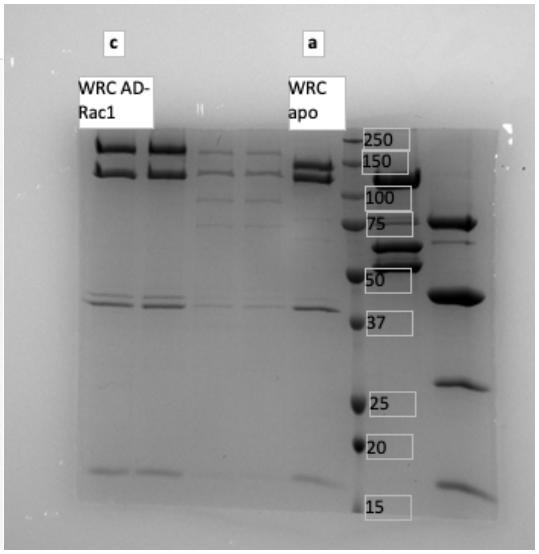


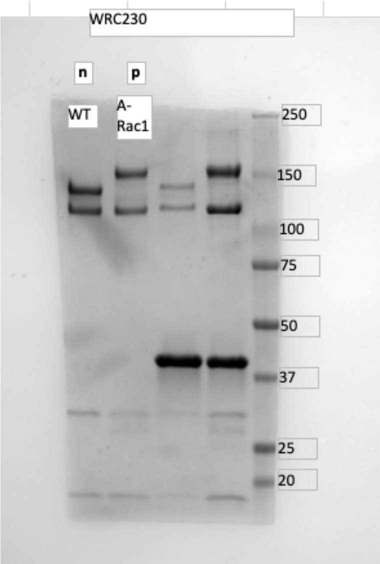


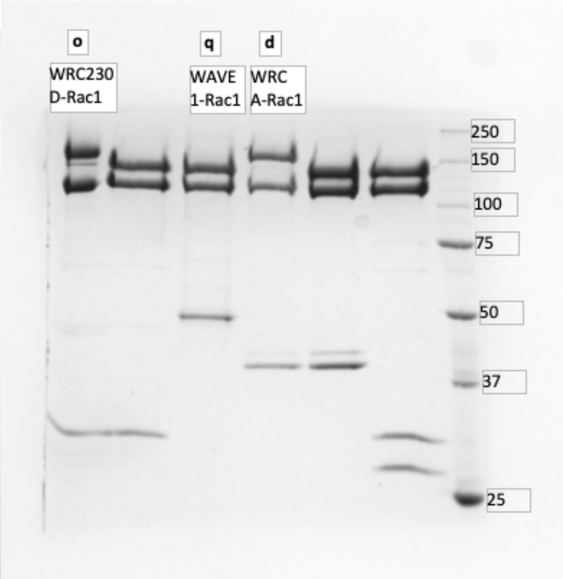

**Uncropped blot images for Supplementary Figure 7c**

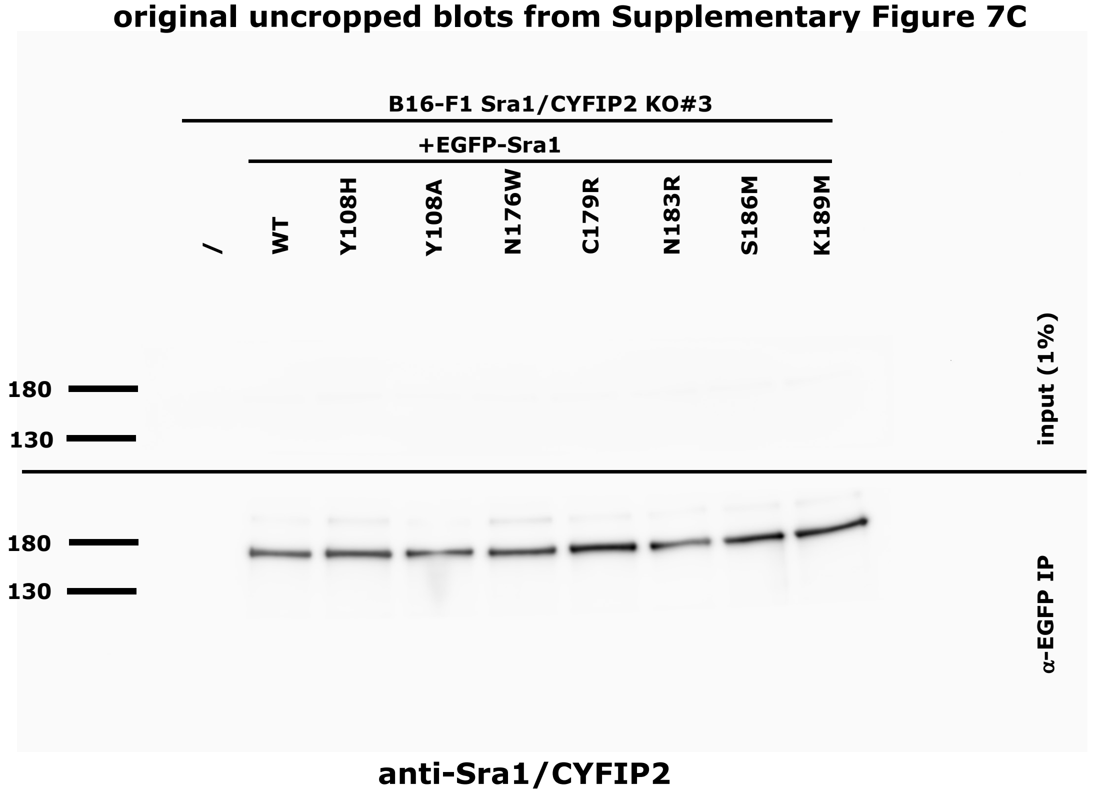


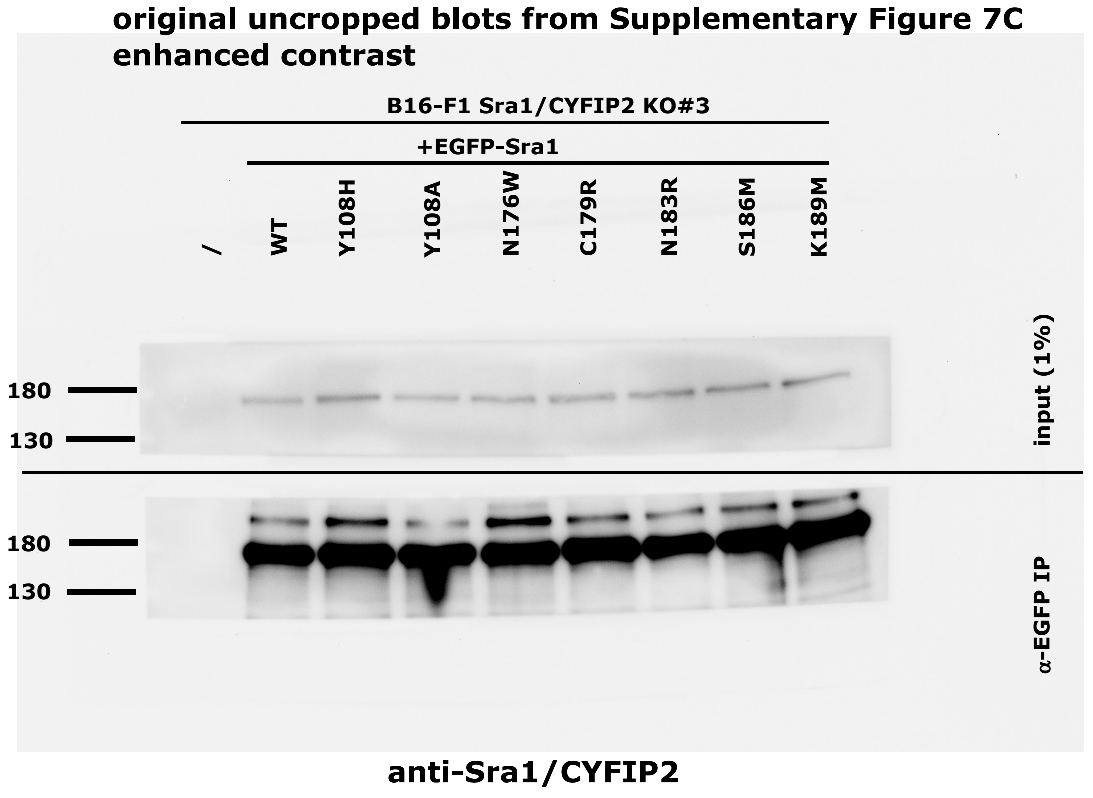

## original uncropped blots from Supplementary Figure 7C

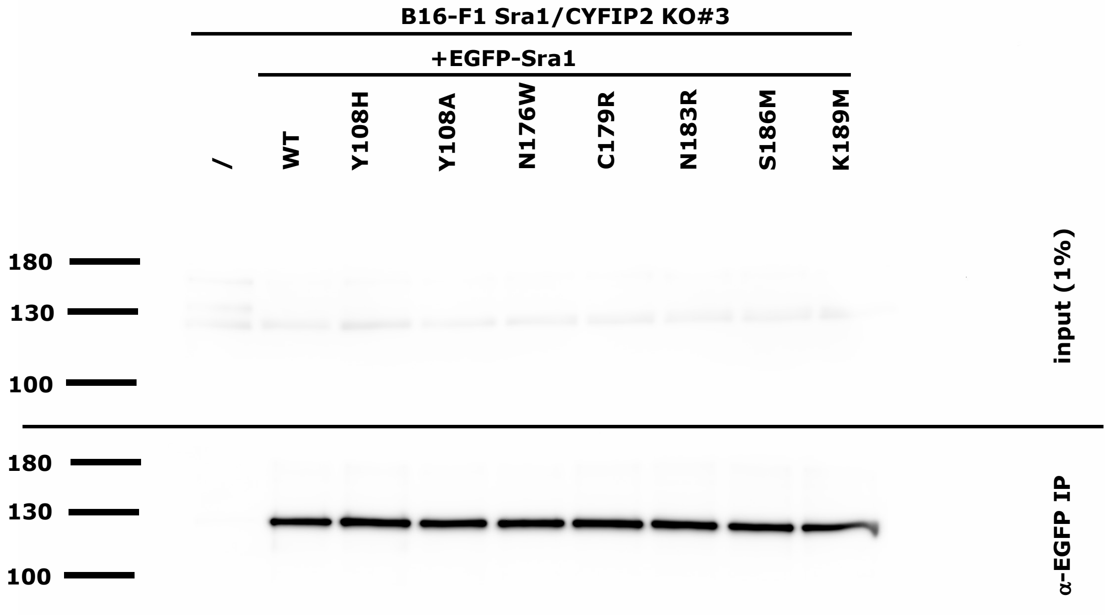


**anti-Nap1**

## original uncropped blots from Supplementary Figure 7C enhanced contrast

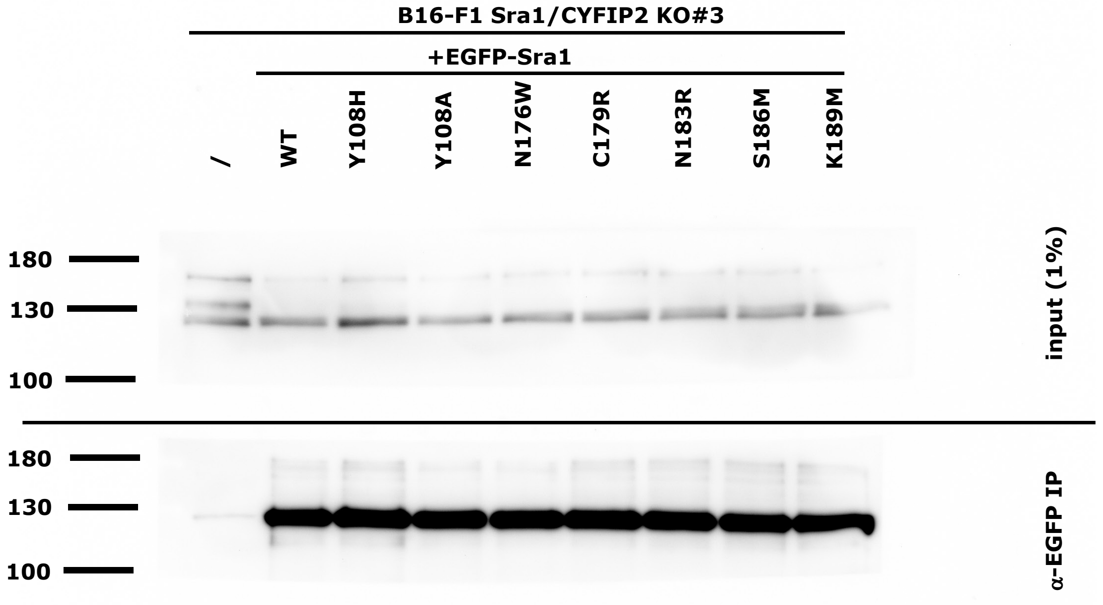


**anti-Nap1**

## original uncropped blots from Supplementary Figure 7C

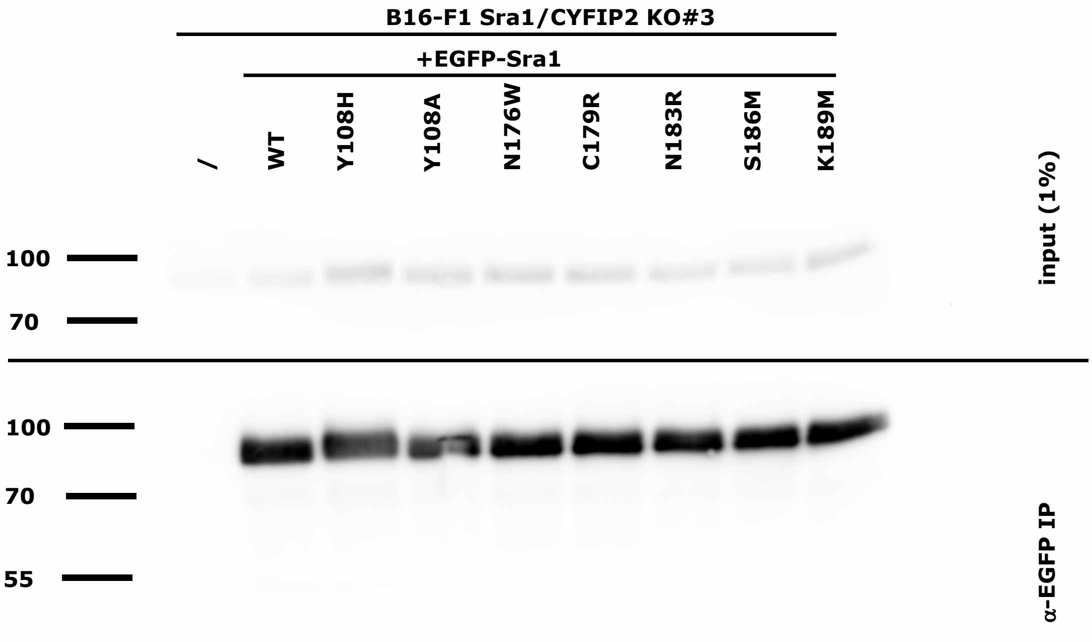


anti-WAVE

## original uncropped blots from Supplementary Figure 7C contrast enhanced

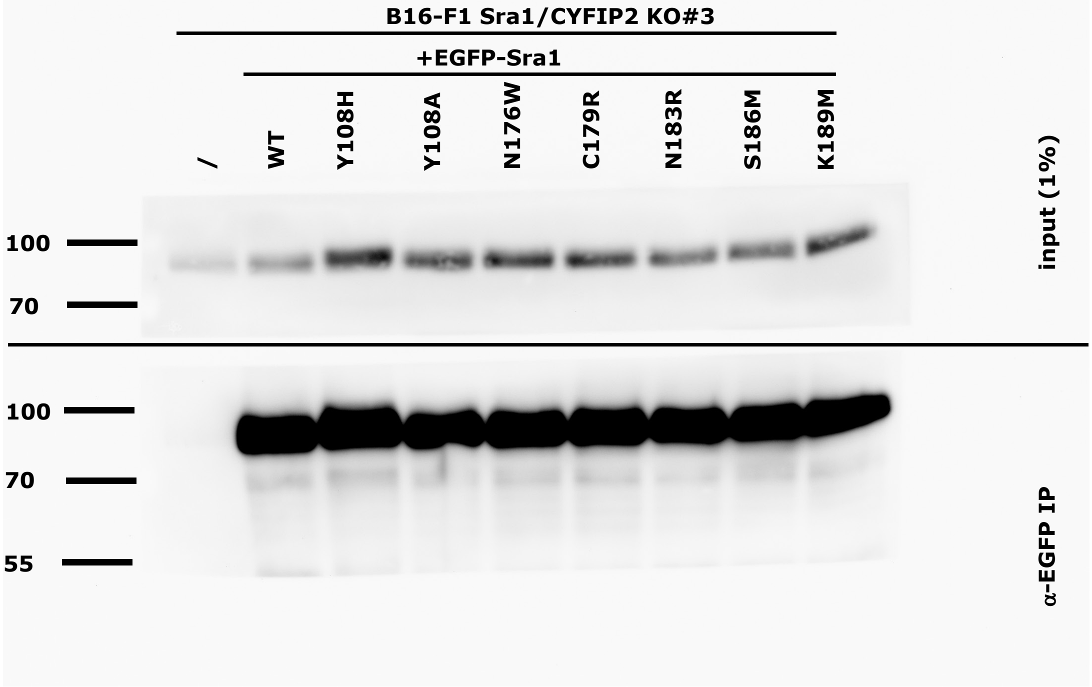


anti-WAVE
