# Supplementary material for: Structures reveal a key mechanism of WAVE regulatory complex activation by Rac1 GTPase
Source: Nat Commun. 2022 Sep 16;13:5444. doi: 10.1038/s41467-022-33174-3 (PMC9481577; doi:10.1038/s41467-022-33174-3)
Supplement: Supplementary file 3 — Description of Additional Supplementary Files [file 41467_2022_33174_MOESM3_ESM.pdf]

Description of Additional Supplementary Files

Supplementary Movie 1. Morph movie showing conformational changes caused by Rac1 binding to the A site leading to WCA release. Movie was made using Pymol based on the structural overlay of WRCD-Rac1 and WRCAD-Rac1. Rac1 (orange) binding to the A site (green) flattens the binding surface, causing a rotation against the tyrosine lock indicated by WAVE1Y151 (blue stick). This rotation in turn destabilizes the tyrosine lock conformation, which leads to the release of the downstream sequences including the W and C helices (red). Structure of the intermediate state was made by combining the structure of WRCAD-Rac (all but the meander and WCA regions of WAVE1) and WRCD-Rac1 (meander and WCA sequences, after aligning the L2 loop of WRCD-Rac1 to WRCAD-Rac1). Structure of the end point was based on the structure of WRCAD-Rac1, with the destabilized structural elements (meander region and WCA) randomly positioned.
