# Supplementary material for: Structures reveal a key mechanism of WAVE regulatory complex activation by Rac1 GTPase
Source: Nat Commun. 2022 Sep 16;13:5444. doi: 10.1038/s41467-022-33174-3 (PMC9481577; doi:10.1038/s41467-022-33174-3)
Supplement: Supplementary file 5 — Reporting Summary [file 41467_2022_33174_MOESM5_ESM.pdf]

nature portfolio

Corresponding author(s): Baoyu Chen; Saikat Chowdhury

Last updated by author(s): Aug 18, 2022

Reporting Summary

Nature Portfolio wishes to improve the reproducibility of the work that we publish. This form provides structure for consistency and transparency in reporting. For further information on Nature Portfolio policies, see our Editorial Policies and the Editorial Policy Checklist.

Statistics

For all statistical analyses, confirm that the following items are present in the figure legend, table legend, main text, or Methods section.

n/a	Confirmed	
☐	☒	The exact sample size (*n*) for each experimental group/condition, given as a discrete number and unit of measurement
☐	☒	A statement on whether measurements were taken from distinct samples or whether the same sample was measured repeatedly
☐	☒	The statistical test(s) used AND whether they are one- or two-sided<br>*Only common tests should be described solely by name; describe more complex techniques in the Methods section.*
☒	☐	A description of all covariates tested
☒	☐	A description of any assumptions or corrections, such as tests of normality and adjustment for multiple comparisons
☐	☒	A full description of the statistical parameters including central tendency (e.g. means) or other basic estimates (e.g. regression coefficient) AND variation (e.g. standard deviation) or associated estimates of uncertainty (e.g. confidence intervals)
☐	☒	For null hypothesis testing, the test statistic (e.g. *F*, *t*, *r*) with confidence intervals, effect sizes, degrees of freedom and *P* value noted<br>*Give P values as exact values whenever suitable.*
☒	☐	For Bayesian analysis, information on the choice of priors and Markov chain Monte Carlo settings
☒	☐	For hierarchical and complex designs, identification of the appropriate level for tests and full reporting of outcomes
☒	☐	Estimates of effect sizes (e.g. Cohen's *d*, Pearson's *r*), indicating how they were calculated

Our web collection on statistics for biologists contains articles on many of the points above.

Software and code

Policy information about availability of computer code

Data collection	single particle cryo-EM data collection: EPU (Thermo Fisher Scientific); chromatography: Unicorn v7 (Cytiva); pyrene-actin polymerization assay: SparkControl v2.2
Data analysis	cryo-EM data processing and model building: RELION v3.0.6, v3.0.7, v3.1; Cryosparc v2; 3DFSC server; UCSF Chimera v1.16; UCSF ChimeraX v1.3; Namdinator; Coot v0.9; Phenix v1.18; Molprobity server; PDB Validation server. Statistical analysis: Prism 6.01.

For manuscripts utilizing custom algorithms or software that are central to the research but not yet described in published literature, software must be made available to editors and reviewers. We strongly encourage code deposition in a community repository (e.g. GitHub). See the Nature Portfolio guidelines for submitting code & software for further information.

Data

Policy information about availability of data

All manuscripts must include a data availability statement. This statement should provide the following information, where applicable:

- Accession codes, unique identifiers, or web links for publicly available datasets
- A description of any restrictions on data availability
- For clinical datasets or third party data, please ensure that the statement adheres to our policy

Cryo-EM reconstructed maps for WRCapo, WRCD-Rac1 and WRCAD-Rac1 were deposited in the Electron Microscopy Data Bank (EMD) under accession IDs EMD-26732, EMD-26733, and EMD-26734 respectively, and corresponding atomic models were deposited in the Protein Data Bank (PDB) with accession IDs 7USC, 7USD, and 7USE respectively.

Human research participants

Policy information about studies involving human research participants and Sex and Gender in Research.

Reporting on sex and gender	n/a
Population characteristics	n/a
Recruitment	n/a
Ethics oversight	n/a

Note that full information on the approval of the study protocol must also be provided in the manuscript.

Field-specific reporting

Please select the one below that is the best fit for your research. If you are not sure, read the appropriate sections before making your selection.

[x] Life sciences [ ] Behavioural & social sciences [ ] Ecological, evolutionary & environmental sciences

For a reference copy of the document with all sections, see nature.com/documents/nr-reporting-summary-flat.pdf

Life sciences study design

All studies must disclose on these points even when the disclosure is negative.

Sample size	Sample size was determined empirically based on prior experiences and capacity of the experimental conditions. We did not use any statistical tests to predetermine sample size, but adhered to established practices in the field. The chosen number of cells was sufficiently large to yield mean values for each experimental replicate that were very similar.
Data exclusions	A subset of single particle cryo-EM images of low quality or heterogeneity were removed using standard procedures during 2D classification and 3D reconstruction.
Replication	All biochemical and cell biological measurements were repeated multiple times (at least twice, except the immunoprecipitation in Supplementary Fig. 7C, which was just performed once) to ensure the same conclusions hold true between repeats. All attempts, excluding those with human mistakes, were successful.
Randomization	For assessment of lamellipodia formation, cells were randomly selected from large data sets. All cells that passed quality control were analyzed equally with no sub-sampling and thus, there was no requirement for randomization. Other experiments do not involve sample randomization.
Blinding	The experimental origin of cell images was blinded to the investigator assessing the efficiency of lamellipodia formation to avoid bias. No other experiments from us could have been blinded.

Reporting for specific materials, systems and methods

We require information from authors about some types of materials, experimental systems and methods used in many studies. Here, indicate whether each material, system or method listed is relevant to your study. If you are not sure if a list item applies to your research, read the appropriate section before selecting a response.

Materials & experimental systems

n/a	Involved in the study
☐	☒ Antibodies
☐	☒ Eukaryotic cell lines
☒	☐ Palaeontology and archaeology
☒	☐ Animals and other organisms
☒	☐ Clinical data
☒	☐ Dual use research of concern

Methods

n/a	Involved in the study
☒	☐ ChIP-seq
☒	☐ Flow cytometry
☒	☐ MRI-based neuroimaging

Antibodies

Antibodies used

anti-Sra1/CYFIP2 (rabbit polyclonal; Steffen et al., 2004; ID: 4955-B) used 1:5000
anti-Nap1 (rabbit polyclonal; Steffen et al., 2004; ID: 4953-B) used 1:5000
anti-WAVE (rabbit polyclonal; Schaks et al., 2018; ID: pkWAVE2) used 1:1000
anti-rabbit IgG HRP-conjugate (goat polyclonal; Dianova, Cat#111-035-045) used 1:5000

Validation

anti-Sra1/CYFIP2 has been validated by gene knockdown and knockout cells (PMID: 14765121, 30393033)
anti-Nap1 has been validated by gene knockdown and knockout cells (PMID: 14765121; https://doi.org/10.1101/2021.06.18.449030)
anti-WAVE has been validated by gene and knockout cells (PMID: 30393033, 32697617)
anti-rabbit IgG HRP-conjugate has been validated by Dianova (https://www.dianova.com/en/shop/111-035-045-goat-igg-anti-rabbit-igg-hl-hrpo-minx-hu/
) by immunoelectrophoresis and ELISA; the antibody reacts with whole molecule rabbit IgG.

Eukaryotic cell lines

Policy information about cell lines and Sex and Gender in Research

Cell line source(s)

Standard, commercial insect cell lines used in this study include Sf9 cells (Thermo Fisher) and Tni (High-Five) cells (Expression System). Mouse melanoma cell B16-F1 Sra-1/Cyfip2 KO#3 were established previously from B16-F1 cells purchased from ATCC (CRL-6323, sex:male) (see Schaks et al., 2018).

Authentication

Commercial insect cells were authenticated based on their stereotypical morphology and growth rate. B16-F1 cells were authenticated by Western Blot for missing Sra-1/Cyfip2 and by their stereotypical morphology (lack of lamellipodia).

Mycoplasma contamination

Commercial insect cells were occasionally tested for lack of mycoplasma contamination by PCR. B16-F1 cell lines (such as the Sra-1/Cyfip2-KO KO#3 line used here) are regularly confirmed to be mycoplasma-free by DAPI staining and PCR, and the parental, wiltype line was authenticated in an official lab screen by local authorities to be mycoplasma-free, of murine origin and to lack any unexpected sequences such as commonly used antibiotic resistance genes or viral sequences.

Commonly misidentified lines
(See ICLAC register)

No commonly misidentified cell lines have been used in this study.
